# Systematic review and meta-analysis of the efficacy and safety of oseltamivir (Tamiflu) in the treatment of Coronavirus Disease 2019 (COVID-19)

**DOI:** 10.1371/journal.pone.0277206

**Published:** 2022-12-01

**Authors:** Basiru Aliyu, Yakubu Egigogo Raji, Hui-Yee Chee, Mui-Yun Wong, Zamberi Bin Sekawi

**Affiliations:** 1 Department of Medical Microbiology and Parasitology, Faculty of Medicine and Health Sciences, Universiti Putra Malaysia, Selangor, Malaysia; 2 Department of Plant Protection, Faculty of Agriculture, Universiti Putra Malaysia, Selangor, Malaysia; 3 Department of Microbiology, Faculty of Sciences, Federal University Birnin Kebbi, Birnin Kebbi, Nigeria; 4 Department of Pathology, Clinical Microbiology Unit College of Health Sciences Ibrahim Badamasi Babangida University Lapai, Lapai Nigeria; Cairo University, EGYPT

## Abstract

Efforts are ongoing by researchers globally to develop new drugs or repurpose existing ones for treating COVID-19. Thus, this led to the use of oseltamivir, an antiviral drug used for treating influenza A and B viruses, as a trial drug for COVID-19. However, available evidence from clinical studies has shown conflicting results on the effectiveness of oseltamivir in COVID-19 treatment. Therefore, this systematic review and meta-analysis was performed to assess the clinical safety and efficacy of oseltamivir for treating COVID-19. The study was conducted according to the PRISMA guidelines, and the priori protocol was registered in PROSPERO (CRD42021270821). Five databases were searched, the identified records were screened, and followed by the extraction of relevant data. Eight observational studies from four Asian countries were included. A random-effects model was used to pool odds ratios (ORs), mean differences (MD), and their 95% confidence intervals (CI) for the study analysis. Survival was not significantly different between all categories of oseltamivir and the comparison groups analysed. The duration of hospitalisation was significantly shorter in the oseltamivir group following sensitivity analysis (MD -5.95, 95% CI -9.91—-1.99 p = 0.003, heterogeneity I2 0%, p = 0.37). The virological, laboratory and radiological response rates were all not in favour of oseltamivir. However, the electrocardiographic safety parameters were found to be better in the oseltamivir group. However, more studies are needed to establish robust evidence on the effectiveness or otherwise of oseltamivir usage for treating COVID-19.

## Introduction

In late December 2019, a new respiratory disease emerged in the Wuhan city of Hubei province, China. Shortly after the emergence of the disease, the causative agent was discovered to be a novel Coronavirus. The virus was provisionally named 2019 novel Coronavirus (2019-nCoV). However, on the 11th of February 2020, the World Health Organisation (WHO), named the disease caused by the 2019-nCOV the “Coronavirus disease 2019” (COVID-19) [1]. Subsequently, the virus was renamed the ‘Severe Acute Respiratory Syndrome Coronavirus -2 (SARS-CoV-2) by the International Committee on Taxonomy of Viruses (ICTV) [2]. SARS-CoV-2 belongs to the Beta-coronavirus genus and Coronaviridae family. The virus is the seventh known human coronavirus (HCoV). The previously discovered HCoVs include; HCoV-HKU1, HCoV-NL63, HCoV-229E, HCoV-OC43, Middle East Respiratory Syndrome Coronavirus (MERS-CoV), and Severe Acute Respiratory Coronavirus (SARS-CoV) [3]. The first four of these viruses cause mild upper respiratory disease while the last two can lead to severe and lethal respiratory illness. SARS-CoV-2 is a large, enveloped coronavirus of approximately 50—200nm in diameter. The virus has a positive-sense single-stranded RNA genome. The viral genome is approximately 30 kb in length and encodes four structural proteins; spike protein (SP), membrane protein (MP), envelope protein (EP), and nucleocapsid protein (NP). COVID-19 has spread throughout the world, causing both asymptomatic and symptomatic infections. The pandemic has continued to be a global public health burden with its consequent economic challenge. Besides, there is yet to be an effective curative therapy although promising vaccine candidates have emerged [4], and many are underway [5]. However, the world is still in search of effective therapeutic agents with a tolerable safety profile for treating COVID-19. As a result, several clinical trials of COVID-19 therapeutics have either been conducted or are ongoing [6]. Some drugs in these trials include hydroxychloroquine (HCQ), remdesivir, oseltamivir, ivermectin, and lopinavir/ritonavir (L/R) amongst others. The results obtained from these trials have either been promising, negative, or conflicting. Oseltamivir is an antiviral drug used for treating influenza A and B viruses [7]. As an ester prodrug oseltamivir is converted to an active intermediate oseltamivir carboxylase, which then acts as an inhibitor of influenza neuraminidase [7]. The drug is effective with a good safety profile for treating influenza virus infection [8]. It has also been suggested that the active site of the spike protein of SARS-CoV [9], has similarities with the neuraminidase of the influenza virus. Thus, indicating that neuraminidase inhibitors can be used for treating SARS-CoV. However, evidence from the existing clinical studies against or in favour of oseltamivir for treating COVID-19 is still a subject of debate. Consequently, this systematic review and meta-analysis was conducted to evaluate the clinical safety and efficacy of this drug for treating COVID-19.

## Materials and methods

### Study design

This study was conducted according to the PRISMA (preferred reporting items of the systematic review and meta-analysis) checklist (S1 File). A priori protocol (S2 File) was designed according to the PRISMA-P checklist (S3 File) for the systematic review and meta-analysis. The protocol was then registered with PROSPERO: CRD42021270821 available at PROSPERO.

### Eligibility criteria

#### Inclusion criteria

All relevant (full-text; observational or randomised controlled trials) articles published in English from the 1st of December 2019 and conducted in any part of the globe were included. The included articles were for studies conducted on patients of all age groups diagnosed with COVID-19 using standard diagnostic guidelines. Additionally, studies included are those that used oseltamivir alone or in combination compared to either usual care (supportive therapy), other drugs (alone or in combination), or placebo.

#### Exclusion criteria

Case reports, letters to the editor, editorials, books, dissertations, review articles, unpublished reports, and conference papers were all excluded. Any published study with incomplete data on the use of oseltamivir and those published in languages other than English were equally excluded.

### Outcomes

The primary outcome assessed in this study is patient recovery from COVID-19 which also refers to survival associated with oseltamivir therapy. Secondary outcomes include: 1) Clinical response defined as the duration of normalisation of signs and symptoms (body temperature, cough, etc) after the initiation of treatment. 2) Virological response is defined as the duration for achieving negative RT-PCR result after the initiation of treatment. 3) Laboratory response is described as the normalisation of the laboratory parameters following treatment. 4) Radiological response specified as the normalisation of X-ray, and/or computer tomographic (CT) results following treatment onset. 5) Duration of hospitalisation is defined as the time from hospital admission to discharge (in days). 6) Safety evaluation is described as monitoring any adverse event; an unfavourable result or negative consequence that happens during or after the use of a drug or other intervention but is not always caused by it, as well as a harmful outcome for which the causal relationship between the intervention and the event is at least conceivable [10].

### Search and selection strategies

The search strategy for this study was conducted to assess all relevant literature citations captured through the application of the search algorithm in selected electronic bibliographic databases. The strategy also included a literature search via hand searching of references of selected (review) articles and conference proceedings. Additionally, an internet search of selected clinical trial registration databases (WHO, EU, US, China), Google Scholar, and Google search was conducted to identify registered clinical trials and more citations.

### Databases

The selected databases that were searched include PubMed, MEDLINE, Scopus, ProQuest, and Embase. The specific search keywords, hits, and specific search dates were detailed in the study protocol (S2 File). However, a sample of the search terms performed on PubMed is as follows ((“Randomise control trial” OR “RCT” OR “Non-randomised control trial” OR “nRCT” OR “Cohort study” OR “Retrospective study” OR “Prospective study” OR “Case series”) AND (“Efficacy” OR “Effectiveness” OR “Effectivity” OR “Safety”) AND (“Oseltamivir” OR “Tamiflu”) AND (“Treatment” OR “Management” OR “Therapy” OR “Cure”) AND (“2019 novel Coronavirus” OR “2019-nCoV” OR “Coronavirus disease 2019” OR “COVID-19” OR “Wuhan coronavirus” OR “Severe acute respiratory syndrome coronavirus 2” OR “SARS-CoV-2”)).

### Data management

The citations obtained from the electronic databases searched were compiled and exported to a web-based systematic review software (Rayyan). All the screening steps (de-duplications, title and abstract screenings, and full-text screening) of the systematic review were conducted on Rayyan [11]. References that met the inclusion-exclusion criteria after the screening steps were exported to Microsoft Excel for data extraction.

### The selection process

Two reviewers carried out the entire screening process blinded to each other, and conflicts were resolved by the third reviewer.

### Data collection process

The extraction of data was conducted after full-text evaluation. The relevant information was extracted from each article included and recorded immediately in the data extraction file. The extraction was carried out by two independent reviewers and two others checked the information.

### Risk of bias (quality) assessment

The relevant articles that met the eligibility criteria were included in the study. The quality of each included article was evaluated based on the Newcastle-Ottawa Scale (NOS) (S4 File) for observational studies [12]. In addition, an assessment of the risk of bias (RoB) was performed using the risk of bias in non-randomised studies of intervention (ROBINS-I) tool [13]. Two independent reviewers conducted the critical appraisal and RoB assessment. While a third reviewer cross-checked the process and resolved the areas of discrepancies.

### Meta-analysis

#### Assessment of heterogeneity

Statistical heterogeneity among the included studies was estimated using the X2 test and I2 statistics. The interpretation of the heterogeneity threshold followed the guide provided in the Cochrane Handbook of Systematic Reviews of Intervention [14]. As follows: An I2 value of 0 to 40% might not be important; 30% to 60% may represent moderate heterogeneity; 50% to 90% may represent substantial heterogeneity; and 75% to 100% considerable heterogeneity [14]. In addition to determine how widely the true effect varies across populations, a 95% prediction interval (PI) estimate was conducted using the comprehensive meta-analysis PIs programme [15].

### Statistical assessment

RevMan5.3 software (Nordic Cochran Centre, Copenhagen, Denmark) provided by Cochrane Collaboration was used for quantitative synthesis. For continuous and dichotomous data, mean difference (MD) and odds ratio (OR) were used respectively for assessing the point estimate, with a 95% confidence interval (CI). The meta-analysis was performed using the random-effects model. Inverse variance or Mantel Hazel methods was used to pool the continuous and dichotomous data respectively.

### Sensitivity analysis

Sensitivity analysis based on the leave-one-out model was done to identify the study that greatly influences the result of the meta-analysis for the primary outcome and the duration of hospitalisation outcome.

### Post-hoc power analysis

Despite the ability of meta-analysis to increase the power for statistical inference, there exist the possibility of the statistical method being underpowered [16]. Leading to the possibility of overestimation or underestimation due to a lack of power and precision in the intervention effect. Thus, having the potential of producing random errors, particularly with meta-analyses of rare events or sparse data, and repetitive testing [16, 17]. Therefore, a post-hoc trial sequential analysis (TSA) was conducted to analyze the dependability and conclusiveness of the available evidence provided in this meta-analysis for the primary outcome and the duration of hospitalisation outcome. The TSA software (version 0.9.5.10 Beta;TSA link was used for the power analysis [18, 19]. The details of how the TSA was conducted for both outcomes are presented in S5 File.

### Publication bias

The publication bias of the primary outcome and duration of hospitalisation was assessed by visuallised inspection of the funnel plot.

### Assessment of quality of evidence

The Grading of Recommendations, Assessment, Development, and Evaluations (GRADE) approach [20] was used to evaluate the quality of evidence of this review using the GRADE pro GDT (guideline development tool) software [21]. The rating of the certainty of the evidence was done on all outcomes (both meta-analysed and narratively synthesised outcomes) [22].

## Results

### Study selection process and characteristics of included studies

**Fig 1:**
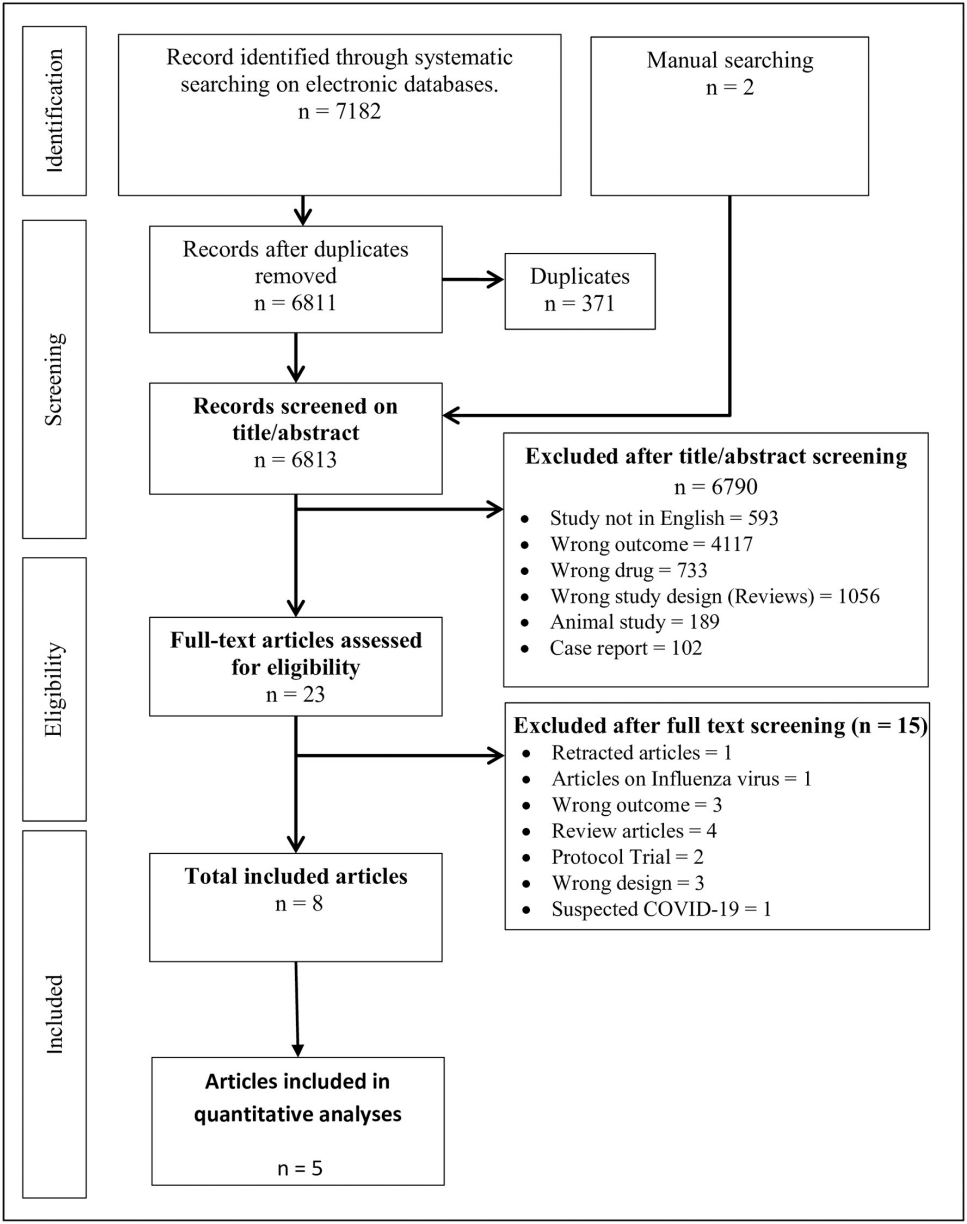
PRISMA flow diagram. Figure shows the entire screening process.

The literature search result from the five selected electronic databases retrieved 7182 citations, 2 citations [23, 24] were found following manual search, and 9 ongoing trials were captured from clinical registry search. The identified citations were deduplicated (371 duplicates) and screened for title/abstract using the Rayyan software for systematic review [11]. After title/abstract screening, 6790 (S1 Table) articles were excluded leaving 23 articles (S2 Table) that were subjected to full–text screening, and only eight [23–30] were included in the study. The screening steps and results are presented in Fig 1. The descriptive characteristics of the studies included in SR&MA are given in Table 1. While the characteristics of the captured ongoing trials are presented in the S3 Table.

**Table 1:** Characteristics of included studies.

Study	Country	Study design	Sample size	Sampled population	Age range/mean (SD)	Sex (%)	Disease severity	Intervention	Control/comparator	Study Outcome
Lee et al., 2020	South Korea	Retrospective Crossectional study	7339	Adult patients with COVID-19	47.1 (± 19 years)	male (40.1)	Severe and non—severe COVID-19	Oseltamivir	Lopinavir/ritonavir, HCQ, Ribavirin, Type 1 interferon, Human immunoglobulin G, and Antibiotics	Death/survival, severity
Ramatillah and Isnaini, 2021	Indonesia	Prospective cohort study	72	ICU admitted COVID-19 patients	19—85 years	male (62.5)	Severe diseae	Oseltamivir, Oseltamivir + CQ, Oseltamivir + HCQ, Favipiravir + Oseltamivir + CQ	Favipiravir +CQ,	Healed/Death
Farrokhpour et al., 2021	Iran	Case control study	104	Severe COVID-19 patients	-	male (65.4)	Severe COVID-19	Two different oseltamivir combinations used as control group+	IVIg 400mg/kg/day for 3–5 days, Infliximab 5mg/kg single dose, and combination of the two	
Haghjoo et al., 2021	Iran	multi-center crossectional study	2365		59.6 ± 16.4	male (54.6)		Oseltamivir 75mg twice daily for 5 days	CQ 500mg twice daily for 1 day then, 250mg twice daily for 5–7 days, HCQ 400mg twice daily for 1 day then, 200mg twice daily for 5–7 days, Lopinavir/ritonavir 200/50mg twice daily, other drugs*	ECG parameters
Vahedi et al., 2020	Iran	Single centre crossectional study	60	COVID-19 in—patients	59.33 ± 14.40 (group I) 57.46 ± 12.74 (group II)	male (41.66)	moderate to severe COVID-19	Oseltamivir 75mg twice daily, HCQ 200mg twice daily, Vamcomycin 1g twice daily, Levofloxacin 500mg daily Meropenem	Azithromycin 250mg daily, Predinisolone 25mg daily, Naproxime 250mg twice daily Lopinavir/Retonavir 200/50mg twice daily	Clinical outcome; Disease progression
Liu et al., 2021	China	Multicenter retrospective cohort study	504	COVID-19 patients	59.5 ± 14.9	Female (48.6)		Oseltamivir	Arbidol, lopinavir/retonavir	in-hospital death, change in lesion size on CT scan
Tan et al., 2021	China	Retrospective cohort study	333	COVID-19 patients	59.52	male (40.8)	mild, moderate, severe, cretical COVID-19	Oseltamivir	Arbidol, corticosteroids,HCQ, Lopinavir/Retonavir	Length of hosp stay, serological level of IgM IgG
Tan et al., 2020	China	Retrospective cohort study	79	COVID-19 patients	50.68 ± 14.912 (0remission grp) 50.33 ± 15.099 (non-remission grp)	male (46.8)	Mild to severe	Oseltamivir (75mg twice daily for 1–3 days, or 3–5 days, r 5–7days)	Non-used-Oseltamivir	Hospitalisation days

+: group1—Oseltamivir + hydroxychloroquine + lopinavir/ritonavir or sofosbuvir, group2—Oseltamivir + hydroxychloroquine + lopinavir/ritonavir or atazanavir + ribavirin or sofosbuvir *: azithromycin 500mg daily for 1 day then, 250mg daily for 5 days; atazanavir/ritonavir 300/100mg daily for 5 days; favipiravir 1600mg twice daily for 1 day then, 600–800mg twice daily for 5 days; remdesivir 200mg daily for 1 day then, 100mg daily for 5–7 days CQ; chloroquine, CT; computerised tomography, g; gram, HCQ; hydroxychloroquine, ICU; intensive care unit, IgG; immunoglobulin G, IgM; immunoglobulin M, kg; kilogram, mg; milligram, SD; standard deviation

### Quality (risk of bias) assessment

**Fig 2:**
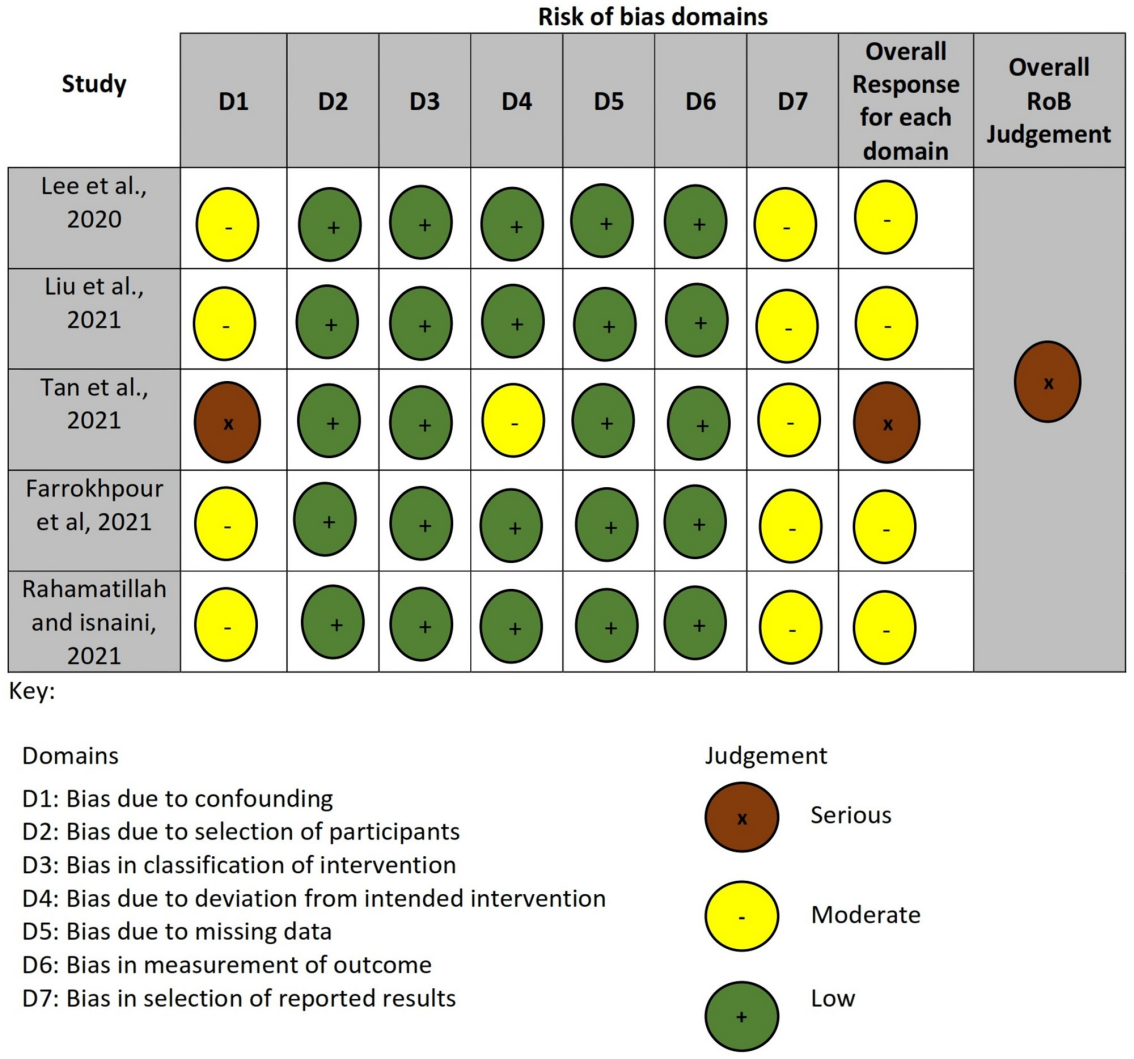
Summary for the ROBINS-I tool. Shows RoB assessment for primary outcome studies.

The result of the quality assessment done using the NOS appraisal tool is presented in Table 2. While Fig 2 presents the result summary for the ROBINS-I tool RoB assessment of the included studies in the primary outcome. Details of the ROBINS-I tool RoB assessment are provided in S6 File.

**Table 2:** Quality (risk of bias) assessment.

	**Selection**				**Comparability**		**Outcome**			**Study Quality**	
Study	Representativeness of exposed cohort	Selection of non-exposed cohort	Ascertainment of exposure	Demonstration that outcome of interest was not present at start of study	The study adjusted for most important risk factors	Study adjusted for other factors	Assessment of outcome	Was follow-up length adequate?	Adequacy of follow-up of cohorts	Total Score	Quality Grading
Rahamatillahl and isnaini, 2021	1	0	1	1	1	1	1	1	1	8	Good
Liu et al., 2021	1	0	1	1	1	1	1	1	1	8	Good
Tan et al., 2020	1	0	1	1	1	1	1	1	1	8	Good
Tan et al., 2021	1	0	1	1	1	1	1	1	1	8	Good
	**Selection**				**Comparability**		**Exposure**			**Study Quality**	
Study	Is the case definition adequate?	Representativeness of the cases	Selection of Controls	Definition of Controls	The study adjusted for most important risk factors	Study adjusted for other factors	Ascertainment of exposure	Same method of ascertainment for cases and controls	Non-Response rate	Total Score	Quality Grading
Farrokhpour et al, 2021	1	1	1	1	1	0	1	1	1	8	Good
**Newcastle-Ottawa Quality Assessment of each included cross-sectional study.**											
	**Selection**				**Comparability**	**Outcome**		**Study Quality**			
Study	Representativeness of	Sample size	Non-respondents	Ascertainment of the exposure (risk factor)	Confounding factors controlled	Assessment of outcome	Statistical test	Total Score	Study Quality Grading		
Lee et al., 2020	1	0	1	2	2	2	1	9	V. Good		
Haghjoo et al., 2021	1	0	1	2	2	2	1	9	V. Good		
Vahedi et al., 2020	1	0	0	2	2	2	1	8	Good		

### Outcomes

**Fig 3:**
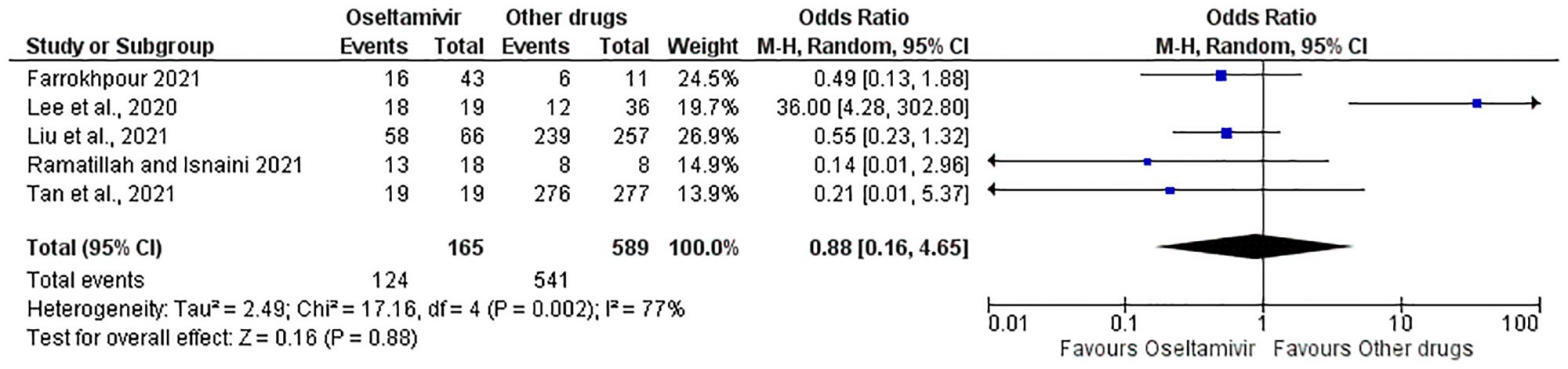
Forest plot. Shows pooled analysis for COVID-19 patients’ survival in the oseltamivir (monotherapy or combination) groups versus other drugs (alone or in combination) treatment.

**Fig 4:**
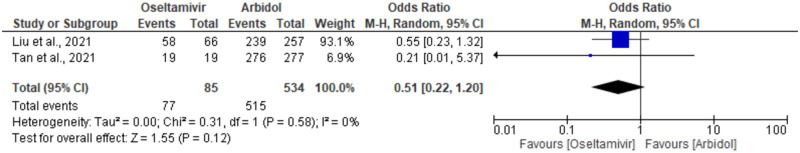
Forest plot. Shows graphical presentation of the meta-analysis comparing the survival of the Oseltamivir group to the Arbidol group.

**Fig 5:**
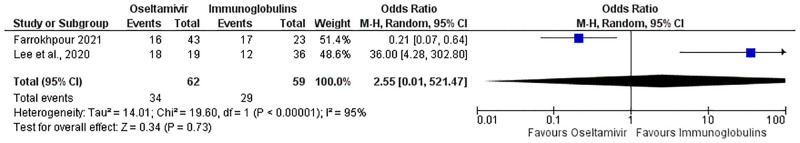
Forest plot. Shows the meta-analysis comparing the survival of the oseltamivir group to the Immunoglobulins group.

**Fig 6:**
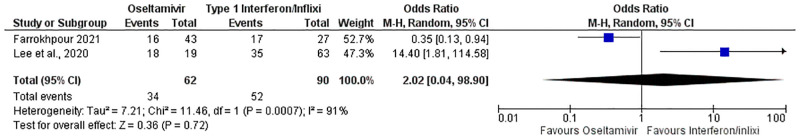
Forest plot. Shows the meta-analysis comparing the survival of the oseltamivir group to the type 1 Interferon/Infliximab groups.

**Fig 7:**
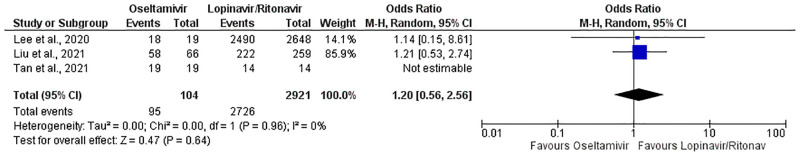
Forest plot. Shows the meta-analysis comparing the survival of the oseltamivir group to the Lopinavir/Ritonavir group.

Primary outcome: Patient recovery from COVID-19 was assessed in five [24, 27–30] of the 8 included studies. Patient recovery was reported as either healed, recovered, or discharged in the five studies. The included studies for this outcome used oseltamivir as monotherapy or in combination and compared it with other drugs as monotherapy or in combination (Table 1). Thus, the meta-analysis for the pooled ORs was done by comparing oseltamivir (alone or in combination) with other drugs (alone or in combination). This revealed an OR of 0.88, 95% CI 0.16—4.65, p = 0.002, heterogeneity [I2] 77%, z = 0.16 (p = 0.88) (Fig 3). From the PI estimation, the true effect was determined to range from 0.003 to 263.8 (S7 File). Furthermore, categories of pooled analyses were done; of oseltamivir monotherapy versus other monotherapy drugs (Figs 4–7). This was done as a form of subgroup analysis since the conventional subgroup analysis could not be used because of the constrain of pair-wise comparison. In some of the studies in the included articles, oseltamivir was compared with more than one drug.

### Sensitivity analysis

**Fig 8:**
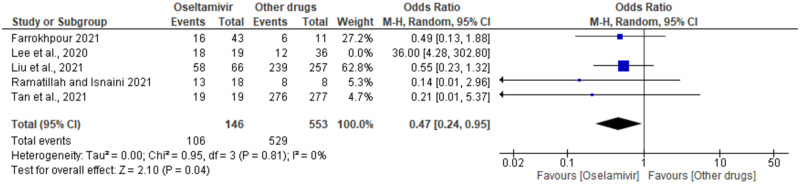
Forest plot. Shows the sensitivity analysis for COVID-19 patients’ survival in the oseltamivir (monotherapy or combination) groups versus other drugs (alone or in combination) treatment.

The sensitivity analysis (leave-one-out) of the overall pooled results was conducted and this showed an OR of 0.47, 95% Cl; 0.24—0.95, p = 0.81, I2 0%, z = 2.10 (p = 0.04) (Fig 8). Here, the PI estimation shows that the effect size is consistent across studies which implies that all studies share a common effect size and there is no dispersion in true effect (S7 File). For the categories comparing oseltamivir to arbidol, and L/R a fixed effect model was used to repeat the analyses (S7 File). The repeated analyses showed no significant difference from the RE model analyses.

### Secondary outcomes

**Fig 9:**
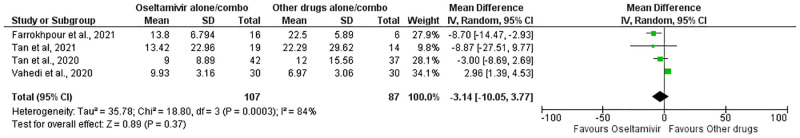
Forest plot. Shows the pooled effect estimate of the duration of hospitalisation of oseltamivir alone/combination compared with other drugs alone/combination.

**Fig 10:**
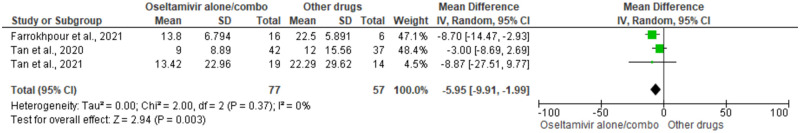
Forest plot. Shows the sensitivity analysis pooled estimate of the duration of hospitalisation of oseltamivir alone/combination vs other drugs.

**Fig 11:**
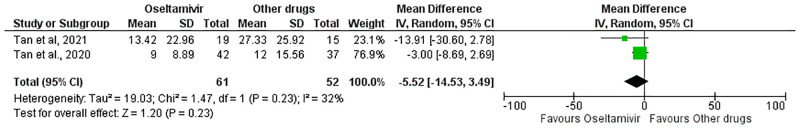
Forest plot. Shows the effect estimate of duration of hospitalisation of oseltamivir monotherapy vs Corticosteroid monotherapy.

**Fig 12:**
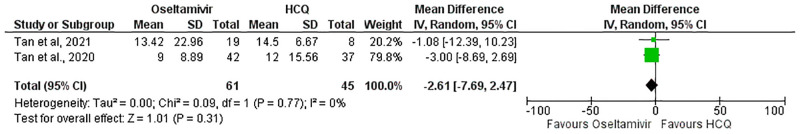
Forest plot. Shows the pooled analysis of the duration of hospitalisation of oseltamivir monotherapy vs HCQ monotherapy.

1. Clinical response associated with oseltamivir treatment; none of the included studies reported this outcome thus, the outcome was not assessed.
2. The virological response associated with oseltamivir therapy; only one study [28] was found to analyse the virological response rate (VRR) of oseltamivir comparing it to four other drugs (L/R, HCQ, corticosteroid, and arbidol). The mean VRR of oseltamivir was 30 days (range: 3—47) as against 28.40 (9—53), 28.94 (1—51), 26.06 (1—74), and 23.43 (6—46) for L/R, HCQ, corticosteroid, and arbidol, respectively.
3. Laboratory response associated with exposure to oseltamivir; only one study was found that evaluated the laboratory tests following treatment with oseltamivir combination therapy compared to other drugs. The study [26] reported the mean change in laboratory parameters for white blood cells (WBC) count, lymphocytes (LYMs) count, platelets (PLTs) count, and C-reactive protein (CRP). The tests were evaluated at baseline (before initiation of treatment) and day—3 results (after treatment commencement). Results showed that there was a significant decrease in the mean CRP concentration in the non-oseltamivir combination therapy group (mean difference [MD]; -55.43, [std. error; 10.82], 95% confidence interval [CI]; -76.63—-34.23, p<0.001) compared to the oseltamivir combination therapy group (MD; -4.64, [8.59], 95% CI; -21.47—12.19, p = 0.589). Also, a statistically significant increase in the mean platelet counts was observed in the two groups. Although the increase was relatively higher in the non-oseltamivir group (MD; 75.44, [12.74], 95% CI; 50.47—100.41, p<0.001) compared to the oseltamivir group, (MD; 51.62 [15.805], 95% CI; 20.64—82.60, p = 0.001). For the WBCs and LYMs counts, the observed mean changes were not statistically significant in both groups.
4. The radiological response; two studies assessed the difference in radiological response between patients exposed to oseltamivir (alone or in combination) and other drugs. One of the studies [26] evaluated the chest CT findings of patients before and after treatment. The chest CT images of the non-oseltamivir treatment group revealed bilateral diffuse ground—glass patchy opacities on admission. However, 10 days after the commencement of treatment (non-oseltamivir), complete resolution of the lesions was observed. On the other hand, the small patchy ground -glass opacities seen in the chest CT of patients on oseltamivir combination treatment on admission transformed into multifocal bilateral consolidations with severe lung involvement 11 days after treatment. The second study [24] looked at the association between treatment (with oseltamivir, arbidol, and L/R) and reduction of lung lesion sizes. The 55 patients who received oseltamivir monotherapy had less average lung lesion reduction compared to the 271 patients that did not (41.18% vs 43.34%).
5. The duration of hospitalisation associated with oseltamivir (alone or in combination) exposure compared with other treatment regimens, was assessed by four studies [23, 26, 28, 29]. Results were pooled for oseltamivir alone/combination compared with other drugs alone/combination. The overall MD was -3.14, 95% CI -10.05—3.77, p = 0.37, heterogeneity I2 84%, p = 0.0003 (Fig 9). The 95% PI was estimated to be -33.0 to 26.7 implying that oseltamivir was clinically effective in reducing the duration of hospitalisation in some studies but not in others (S7 File) However, when the study [26] with the highest weight was excluded, the overall effect was MD -5.95, 95% CI -9.91—-1.99 p = 0.003, heterogeneity I2 0%, p = 0.37 (Fig 10). The estimated PI shows that all studies share a common effect size following the removal of one of the studies (S7 File). Results were also pooled for oseltamivir monotherapy compared to other monotherapies; Oseltamivir versus corticosteroids (Fig 11), and oseltamivir compared to HCQ (Fig 12).
6. Safety evaluation (adverse event); only one study was found that evaluated the safety of Oseltamivir therapy by monitoring the electrocardiographic (ECG) parameters. The study [25] looked at the incidences of corrected QT (QTc) prolongation (QTc ≥500 milliseconds [ms] and ΔQTc ≥60 ms) and torsade de points (Tdp) ECG parameters. The study compared these ECG parameters between those exposed to oseltamivir and other drugs (Table 1). Of the 18 patients treated with oseltamivir monotherapy, none had QTc prolongation of more than 500 ms and Tdp but three (16.7%) had ΔQTc ≥60 ms. However, for those treated with oseltamivir and azithromycin (AZM) combination (103 patients), eight (7.8%) developed QTc ≥500 ms and 10 (9.7%) had ΔQTc ≥60 ms. Still, none in this group developed Tdp. With HCQ monotherapy (41/350 had QTc ≥500 ms, 63/350 had ΔQTc ≥60 ms and none had Tdp). As against 155/1080, 237/1080, and 4/1080 that developed QTc ≥500 ms, ΔQTc ≥60 ms, and Tdp, respectively when AZM was added to HCQ. For the single–drug L/R combination, 27/483, 38/483, developed QTc ≥500 ms and ΔQTc ≥60 ms, respectively and none developed Tdp. Also, when AZM was added to L/R, 25, 52, and 5 of 206 had QTc ≥500 ms, ΔQTc ≥60 ms, and Tdp, respectively.

### Post-hoc power analysis

**Fig 13:**
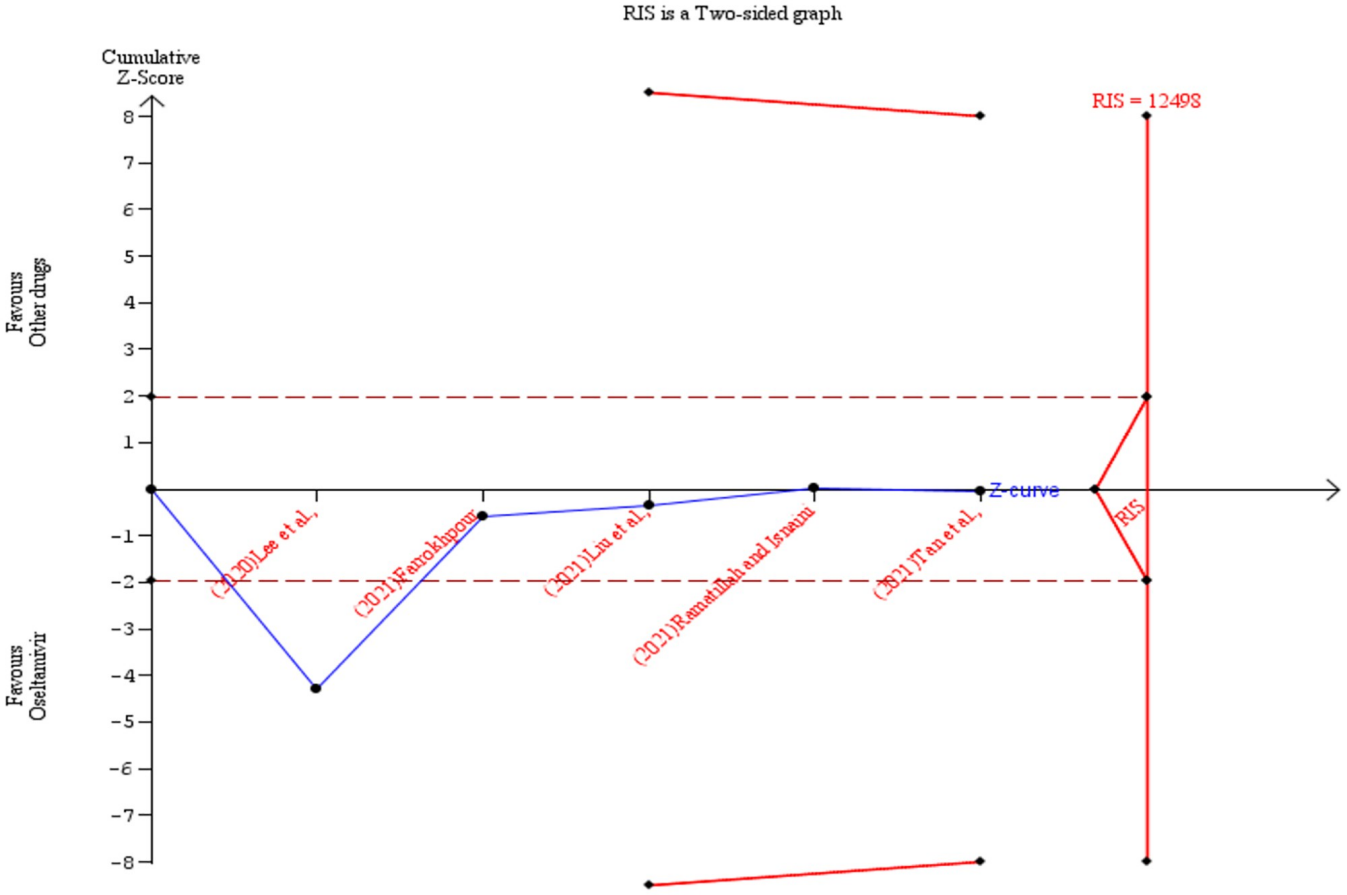
Post-hoc analysis. Shows the trial sequential analysis result comparing the survival of COVID-19 patients treated with oseltamivir versus other drugs.

**Fig 14:**
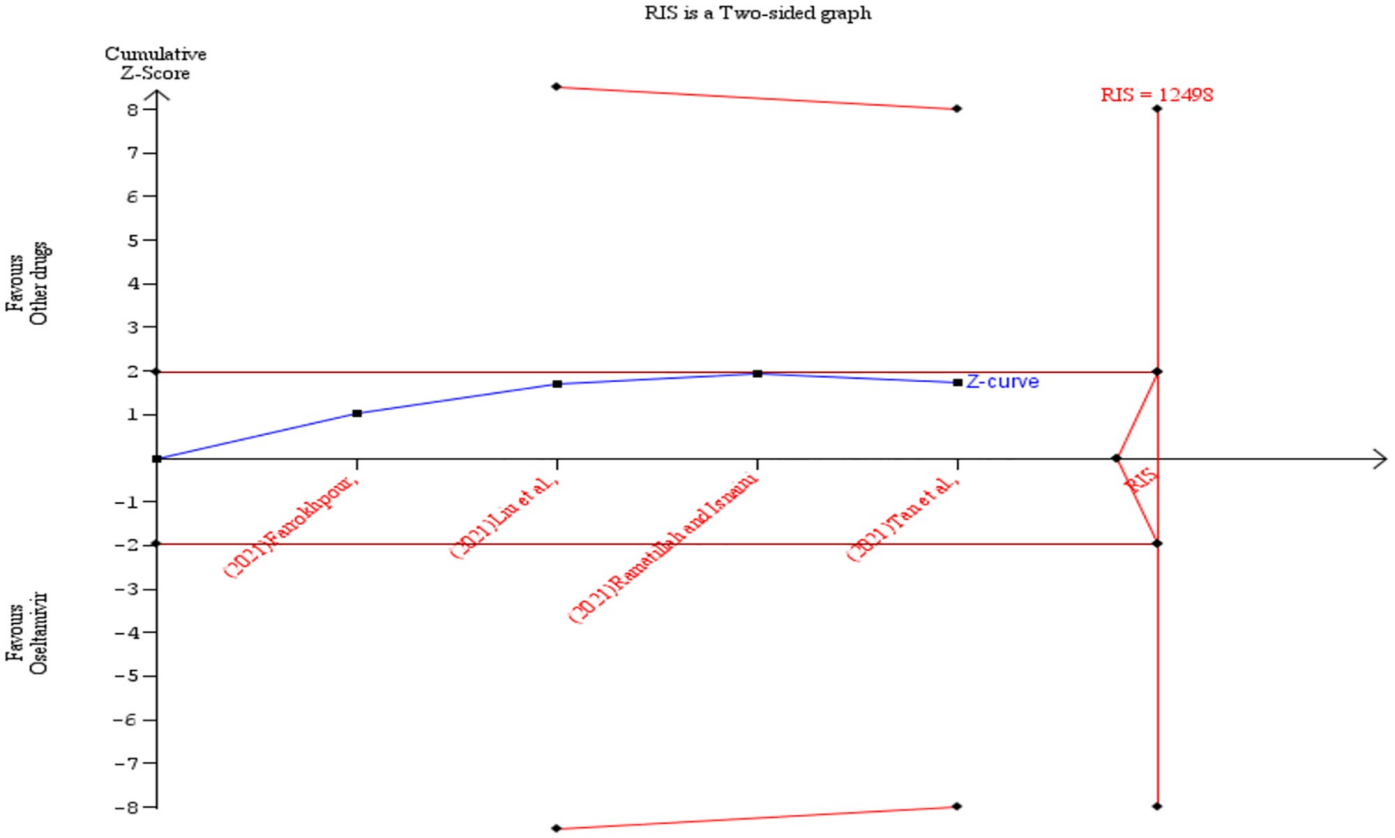
Post-hoc analysis. Shows the trial sequential analysis result comparing the survival of COVID-19 patients treated with oseltamivir versus other drugs after removing one study.

**Fig 15:**
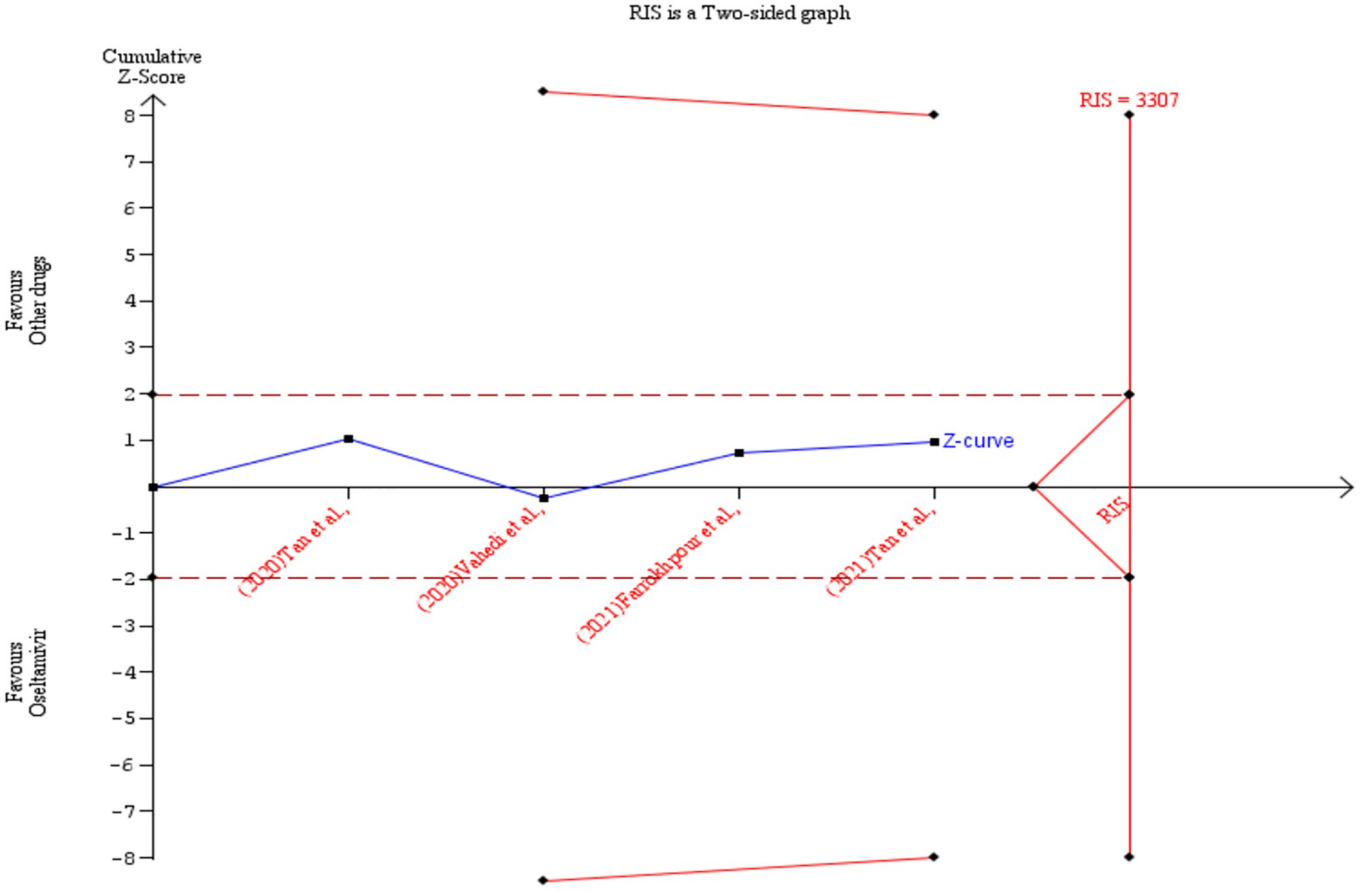
Post-hoc analysis. Shows the trial sequential analysis result comparing the duration of hospitalisation of COVID-19 patients treated with oseltamivir versus other drugs.

To achieve a power of 80% the required information size (RIS) was calculated as 12498 (sample size) for the primary outcome (patient survival). According to the TSA result, the z-score curve did not cross any boundary and it is still within the ‘not statistically significant zone’. This suggests that the observed effect while in favour of oseltamivir was not definitive and that additional studies are needed before any conclusions can be drawn (Fig 13). Further details of the TSA results are presented in the S5 File. The TSA was also conducted by removing one of the studies [26] and showed similar results but with 0% heterogeneity (Fig 14). Additionally, S8 File shows results of the TSA conducted using odds ratio instead of Peto’s odds ratio. For the duration of hospitalisation outcome, the RIS was estimated as 3307 (cumulative sample size). The TSA revealed a z-score curve within the ‘not statistically significant zone’ without crossing any boundary. This indicates the need for more studies before conclusions are made (Fig 15). The details of the TSA are provided in the S9 File.

### Publication bias

Publication bias was assessed for the patient recovery and duration of hospitalisation outcomes. Although there are only five and four included studies in the respective outcomes (while the rule of thumb requires ≥10 studies for a funnel plot), the funnel plot for the studies in the two outcomes shows a nearly symmetrical distribution of the individual studies around the point estimates (S10 File). Thus, indicating that publication bias is not likely.

### Assessment of quality of evidence

The quality of evidence for the results of the evaluated outcomes (primary and secondary) is presented in the summary of the findings table (Table 3). A detailed explanation of the GRADE evaluation is described in the S10 File. The critical assessed outcome had a “moderate” quality of evidence. Other outcomes that were evaluated as important had quality of evidence ranging from “high to very low”. Thus, the overall GRADE assessment was recommended as a “moderate” quality of evidence.

**Table 3:** Summary of findings table for GRADE approach quality of evidence assessment for evaluated outcomes. Population: hospitalised COVID-19 patients, Setting: hospital, Intervention: Oseltamivir alone or in combination, Comparison: supportive care, other drugs, or placebo

Certainty assessment							Nº of patients		Effect		Certainty	Importance
Nº of studies	Study design	Risk of bias	Inconsistency	Indirectness	Imprecision	Other considerations	Oseltamivir alone/combination	other drugs alone/combination	Relative (95% CI)	Absolute (95% CI)		
**Patient recovery (survival) from COVID-19 associted with oseltamivir therapy: Using Oseltamivir alone/combination Vs Other drugs alone/conmbination**												
5	observational studies	serious^a^	not serious^b^	not serious	not serious	none^c^	124/165 (75.2%)	541/589 (91.9%)	**OR 0.88** (0.16 to 4.65)	**1 fewer per 100** (from 25 fewer to 6 more)	***0 Moderate	CRITICAL
								93.0%		**1 fewer per 100** (from 22 fewer to 5 more)		
**Virological response rate (follow-up: 74 days; assessed with: Days)**												
1	observational studies	serious^d^	not serious	not serious	serious^d^	none^c,d^	In this study, oseltamivir has a mean duration of 30 days to negative conversion as against 28.40, 28,94, 26.06, and 23.43 for L/R, HCQ, corticosteroid, and arbidol respectively.				**00 Low	IMPORTANT
**Laboratory response**												
1	observational studies	serious^d^	not serious	not serious	serious^d^	none^c,d^	In this study, the assessment of most of the laboratory parameters were in favour of the comparison groups when compared to oseltamivir group.				**00 Low	IMPORTANT
**Radiologic response (assessed with: CT lung lesions)**												
2	observational studies	serious^d^	serious^d^	not serious	serious^d^	none^c,d^	The two studies reported conflicting results on the resolution of the computerised tomographic (CT) lesions observed. One of the studies (Tan etal., 2020) reported lung lesion reduction in favour of the oseltamivir group. While the other studies revealed worsening lung lesion in the oseltamivir group.				*000 Very low	IMPORTANT
**Duration of Hospitalisation associated with Oseltamivir treatment**												
4	observational studies	serious^e^	not serious	not serious	serious^f^	none^c,d^	107	87	-	MD **3.14 Days fewer** (10.05 fewer to 3.77 more)	**00 Low	IMPORTANT
**Safety evaluation; adverse event (assessed with: monitoring of electrocardiographic (ECG) parameters (in milliseconds))**												
1	observational studies	not serious	not serious	not serious	not serious	none^c,d^	The monitored ECG parameters showed results in favour of the oseltamivir group when compared to other drugs.				****High	IMPORTANT

**CI:** confidence interval; **MD:** mean difference; **OR:** odds ratio

**Explanations**

^a^. One study (Tan et al., 2021) had serious RoB response in D1 of ROBINS-I tool assessment.

^b^. I2 is 74% with P value of 0.004. However, in sensitivity analysis after the removal of one study the I2 reduced to 0% with a P value of 0.81.

^c^. Not assessed

^d^. Refer to S10 File for more information

^e^. Three studies (Tan et al., 2020, Vahedi et al., 2020 & Tan et al., 2021) had serious RoB responses in D1 of the ROBINS-I Rob assessment.

^f^. Two out of the four studies have the lower boundaries of their CI below the threshold. While one of the studies has the entire CI and point estimate below the threshold.

## Discussion

COVID-19 has continued to ravage the world with tremendous public health and economic consequences. Likewise, efforts are still ongoing toward finding an effective therapeutic agent for its treatment. Thus, oseltamivir a widely used anti—influenza drug has been repurposed in many studies for treating COVID-19. The use of oseltamivir in treating COVID-19 is connected to the fact that as a neuraminidase inhibitor, the drug is likely to inhibit SARS-CoV by targeting the S1 protein activity. Zhang et al., in their study, have shown that there is a similarity between the active centre of the influenza virus neuraminidase and SARS-CoV’s S1 protein [9]. This evidence, therefore, has prompted the use of oseltamivir in treating SARS-CoV, MERS-CoV, and SARS-CoV-2. However, the use of oseltamivir for treating COVID-19 has produced conflicting results. Hence, the need for a systematic review to evaluate the effectiveness of this drug for treating COVID-19.

The studies included in this review consist of patients with all spectrums of COVID-19 severity from mild to severe and critical disease with different drug regimens. However, in terms of patients’ survival, the meta-analysis showed no statistical significance. Although the results are in favour of oseltamivir treatment.

Nevertheless, the lack of statistically significant results obtained may not necessarily connote the absence of clinical relevance. Evidence from the result of the meta-analysis can be interpreted as an increased likelihood of a true treatment effect in the population as indicated by the PI estimation. This may in turn imply clinical significance in favour of the oseltamivir group. Thus, effect size estimates between groups might be of relevance in clinical decision-making regardless of the statistical significance [31]. Therefore, the consideration of the possibility of clinical significance is important and has been emphasised in other studies [32]. Based on the TSA result, the evidence provided in this study for the primary outcome is insufficient to confirm or rule out the effectiveness of oseltamivir in improving COVID-19 patients’ survival. However, based on GRADE evaluation there is moderate confidence that the estimated effect (of the survival outcome) is likely close to the true effect.

This review also provided information on the virological cure rate for the use of oseltamivir in treating COVID-19. The finding of this review revealed that patients treated with oseltamivir had a longer duration of viral clearance compared to the controls. Although, the GRADE assessment showed limited confidence in the quality of the evidence. However, some systematic reviews that pooled results comparing corticosteroids [33], or HCQ [34] to controls found no statistically significant differences between the groups in evaluating the VRR. Similarly, regarding laboratory response evaluation, this review has shown that oseltamivir does not have any significant effect on the normalisation of CRP, PLTs, WBC, and LYMs. However, it is worth mentioning here that the non—oseltamivir drug combination used as a control was not outlined in the study [26]. Additionally, the study [26] did not give the prescription details on the administration of the drugs in the two groups. Thus, there is a high chance of prescription bias, which will hinder a fair comparison in the study [26] and with other similar studies. Also, the confidence in the quality of the evidence for this outcome is adjudged as low. Furthermore, this systematic review also assessed the radiological response of exposure to oseltamivir. The result obtained showed that the oseltamivir treatment group had lower CT lesion reduction compared to the comparison groups. With a likelihood of an increased lesion mass associated with oseltamivir use. Although there is very little confidence in the quality of this result.

In terms of the duration of hospitalisation, the meta-analysis showed that oseltamivir demonstrated a non-significant reduction in the duration of hospital stay. However, the observed decrease in hospital stay revealed from this meta -analysis, might be of both clinical and economic relevance. Considering the importance of the duration of hospital stay on the patients, patient relatives, health facility, and healthcare workers. Also, most of the studies included in this review were conducted in developing countries. Thus, the importance of the reduction in terms of economic, physical, emotional, and health burdens due to the shorter duration of hospital stay on the above-mentioned cannot be overemphasized. In addition, the TSA result shows that the existing evidence on the duration of hospitalisation can not be considered conclusive. Equally, the GRADE evaluation revealed that there may be a substantial difference between the estimated effect and the true effect.

In this review also, information was provided on the safety of oseltamivir use in treating COVID-19. This review has indicated that oseltamivir has a relative safety profile with high confidence in the quality of the evidence. It was observed that the risk of QTc prolongation is lower in oseltamivir monotherapy. Also, this study has shown that the combination of AZM with either oseltamivir, HCQ, or L/R, increases the risk of QTc prolongation and Tdp incidence. This result agrees with the results of previous studies that have linked HCQ monotherapy or in combination with AZM in COVID-19 patients, to frequent QTc prolongation and/or development of cardiac arrhythmias [35, 36]. However, there have been conflicting reports from some previous systematic reviews of the increased risk of QTc prolongation or torsadeogenicity of using HCQ, AZM (alone), or their combination in COVID-19 patients. A systematic review observed an increased risk of QTc prolongation with the use of AZM and HCQ [33]. In another study [37] however, no statistically significant difference was found in the treatment groups compared to the control groups.

This study is the first systematic review and meta—analysis to evaluate the efficacy and safety of oseltamivir therapy in COVID-19 patients. Also, all studies included in this review are of good quality and the assessed study outcomes are all pertinent in the evaluation of drug effectiveness. In addition, there is moderate confidence in the overall quality of evidence. However, only a few studies were included, all included studies are observational, all were conducted in Asia, and with reported inconsistent results. Including a limited number of studies in a systematic review, maybe deemed inadequate in providing robust evidence for inference on a general population. The limitation of observational studies in assessing causal inferences may also hamper the study’s strength. The inclusion of RCTs known for demonstrating causality would have given more strength to this review. An additional limitation of this study is the fact that the studies included are from one region of the world. Thereby making it difficult for the generalisation of the result to infer other regions of the world. Overall, therefore, the interpretation of the result of this review should be considered within the framework of these limitations.

## Conclusion

The evidence obtained from this study has pointed out some benefits of oseltamivir in the treatment of COVID-19. The study has also highlighted areas of concern in the effectiveness of the drug in COVID-19 treatment. However, the evidence provided in this review is far from being conclusive. A clear-cut decision on the effectiveness or otherwise of the drug should be done with caution. More studies (especially RCTs) are needed for a shred of robust evidence in favour or against the efficacy of oseltamivir in the treatment of COVID-19.

## Supporting information

S1 File PRISMA checklist. (DOCX)

S2 File Study protocol. (DOCX)

S3 File PRISMA—P checklist. (PDF)

S4 File Adopted NOS. (DOCX)

S5 File Summary TSA- using Peto’s OR. (DOC)

S6 File ROBINS-I assessment tool procedure. (DOCX)

S7 File FE model meta-analysis for primary outcome. (DOCX)

S8 File Summary TSA—OR. (DOCX)

S9 File Summary TSA for duration of hospitalisation outcome. (DOCX)

S10 File GRADE approach evaluation for estimated outcomes. (DOCX)

S1 Table Articles excluded after tittle/abstract screening. (XLSX)

S2 Table Articles screened for full-text. (DOCX)

S3 Table Ongoing clinical trials for oseltamivir. (DOCX)
