# Supplementary material for: Systematic review and meta-analysis of the efficacy and safety of oseltamivir (Tamiflu) in the treatment of Coronavirus Disease 2019 (COVID-19)
Source: PLoS One. 2022 Dec 1;17(12):e0277206. doi: 10.1371/journal.pone.0277206 (PMC9714710; doi:10.1371/journal.pone.0277206)
Supplement: S2 File — (DOCX) [file pone.0277206.s002.docx]

S1 File: Study protocol

**Tittle:** Protocol for A systematic Review and Meta-analysis of the Efficacy and Safety of Oseltamivir (Tamiflu) in the Treatment of Coronavirus disease 2019 (COVID19).

**Registration:** Registered in PROSPERO

Authors: Basiru Aliyu ^1,3^ , Yakubu Egigogo Raji ^1,4^, Chee Hui Yee^1^, Mui-Yun Wong^2^ and Zamberi Bin Sekawi ^1,^*

1. Department of Medical Microbiology and Parasitology, Faculty of Medicine and Health Sciences, Universiti Putra Malaysia 1; [rajiegigogoy@ibbu.edu.ng](mailto:rajiegigogoy@ibbu.edu.ng) (Y.R), [bamailato67@gmail.com](mailto:bamailato67@gmail.com) (B.A), [cheehy@upm.edu.my](mailto:cheehy@upm.edu.my) (C.H.Y), and [zamberi@upm.edu.my](mailto:zamberi@upm.edu.my) (Z.S)
2. Department of Plant Pathology, Faculty of Agriculture, Universiti Putra Malaysia; [muiyun@upm.edu.my](mailto:muiyun@upm.edu.my) (M.W)
3. Department of Microbiology, Faculty of Sciences, Federal University Birnin Kebbi, Nigeria; [basiru.aliyu@fubk.edu.ng](mailto:basiru.aliyu@fubk.edu.ng) (B.A)
4. Department of Pathology, Clinical Microbiology Unit College of Health Sciences Ibrahim Badamasi Babangida University Lapai Nigeria; [rajiegigogoy@ibbu.edu.ng](mailto:rajiegigogoy@ibbu.edu.ng) (Y.R)

***** Correspondence: [zamberi@upm.edu.my](mailto:zamberi@upm.edu.my) ; Tel.: +600389410130

**Authors’ contributions:** Conception of research idea (ZS), Literature review (BA and YR), Research protocol design (YR), Study appraisal (ZS, CHY, and MW), Data extraction (BA, YR, and ZS), Data analysis and interpretation of results (ZS, CHY, MW, BA, and YR), Manuscript drafting (BA and YR), and review of the initial and final draft of the manuscript (ZS, CHY, and MW)**.**

**Source of Support:** Universiti Putra Malaysia

**Sponsor:** Universiti Putra Malaysia

**Role of sponsor:** The sponsor did not play any role in the development of the study protocol.

**Introduction**

The emergence of ‘severe acute respiratory syndrome-related coronavirus-2 (SARS-CoV-2) the causative agent of Coronavirus disease 2019 (COVID-19) has presented numerous challenges. Spreading across the globe causing unprecedented public health and economic challenges. In addition, the world is still racing for an effective therapeutic agent for treating the disease. This search includes efforts at developing new drugs or repurposing the existing ones. Some of the drugs being used as repurpose agents include hydroxychloroquine, remdesivir, ivermectin, lopinavir-ritonavir and oseltamivir amongst others.

The antiviral medication oseltamivir is used to treat influenza A and B viruses [[1](#_ENREF_1)]. As an ester prodrug, oseltamivir is transformed to the active intermediate oseltamivir carboxylase, which subsequently functions as a neuraminidase inhibitor [[1](#_ENREF_1)]. In the treatment of influenza virus infection, the medication is effective and has a good safety profile [[2](#_ENREF_2)]. It has also been suggested that the active site of the SP SARS-CoV [[3](#_ENREF_3)], has similarity with the neuraminidase of Influenza virus. As a result, neuraminidase inhibitors could be employed to treat SARS-CoV infections. As a result of this, a few clinical trials evaluating the efficacy of oseltamivir in treating COVID-19 have been conducted or are presently being conducted. Consequently, there is a need for a systematic review and meta-analysis to evaluate the clinical safety and efficacy of this drug in the treatment of COVID-19.

**Objective**

The aim of this study is to systematically assess the efficacy and safety of Oseltamivir (Tamiflu) alone or in combination with any drug in the treatment of patients diagnosed of COVID-19 using published literature.

Specific objectives:

1. Clinical response or cure; duration of normalisation of signs and symptoms (body temperature, cough) after initiation of treatment.
2. Virological response or cure; duration for achieving negative RT-PCR result.
3. Laboratory biochemical response; normalisation of complete blood count results, C-reactive protein etc.
4. Radiological response; normalisation of x-ray, computer tomographic results.
5. Duration of hospitalisation; time from hospital admission to discharge.
6. Adverse events and reactions of treatment (safety and tolerability)

**Methods**

- Inclusion criteria
- Study location: the study will assess all accessible published full article across the globe.
- Context: the study will assess all observational (cohort, case series, prospective and retrospective) studies, randomised control trial and Quasi experimental studies.
- Time period: all published articles from 1^st^ December 2019
- Language of Publication: English
- Participants: all patients that meets the diagnostic criteria of COVID-19 according to the standard guideline stipulated or adopted in the country of the research.
- Intervention: the use of oseltamivir alone or in combination with any other therapeutic agent in comparison with standard care alone or placebo.
- Age: any age range.
- Exclusion criteria
  - - Case reports, letters to editor, editorials, books, dissertations, review articles, unpublished reports, and conference papers.
    - Any published study with incomplete data on the use of oseltamivir.
    - Articles published in languages other than English.

Search strategy

The search strategy for this study will be to assess all relevant literature citations captured through the application of the search algorithm in selected electronic bibliographic databases. The strategy will also include grey literature search via hand searching of references of selected (review) articles and conference proceedings. Additionally, an internet search of selected clinical trial registration databases (WHO, EU, US, China) will be carried out including Google scholar and Google search using the same algorithm in the bibliographic database search.

**Databases**

The Selected databases to be used include PubMed, MEDLINE, Scopus, Science Direct, ProQuest, and ASEAN Citation Index. The electronic search strategy will be as follows:

**Library/Database:** PubMed

**Date of Search:** March 12, 2021 **(first search date)**

**Search String:** ((“Randomise control trial” OR “RCT” OR “Non-randomised control trial” OR “nRCT” OR “Cohort study” OR “Retrospective study” OR “Prospective study” OR “Case series”) AND (“Efficacy” OR “Effectivenes” OR “Effectivity” OR “Safety”) AND (“Oseltamivir” OR “Tamiflu”) AND (“Treatment” OR “Management” OR “Therapy” OR “Cure”) AND (“2019 novel Coronavirus” OR “2019-nCoV” OR “Coronavirus disease 2019” OR “COVID-19” OR “Wuhan coronavirus” OR “Severe acute respiratory syndrome coronavirus 2” OR “SARS-CoV-2”))

**# Hits:**  7 (hits for the first search).

**Hit result of the repeat search:** 7 (searched on 17/7/2021).

**Library/Database:** MEDLINE (through EBSCOhost)

**Date of Search:** March 12, 2021 **(first search date)**

**Search String:**  ((“Randomise control trial” OR “RCT” OR “Non-randomised control trial” OR “nRCT” OR “Cohort study” OR “Retrospective study” OR “Prospective study” OR “Case series”) AND (“Efficacy” OR “Effectivenes” OR “Effectivity” OR “Safety”) AND (“Oseltamivir” OR “Tamiflu”) AND (“Treatment” OR “Management” OR “Therapy” OR “Cure”) AND (“2019 novel Coronavirus” OR “2019-nCoV” OR “Coronavirus disease 2019” OR “COVID-19” OR “Wuhan coronavirus” OR “Severe acute respiratory syndrome coronavirus 2” OR “SARS-CoV-2”))

**# Hits:**  231 (hits for the first search).

**Hit result of the repeat search:** 248 (searched on 17/7/2021).

**Library/Database:** Scopus

**Date of Search:** March 12, 2021 **(first search date)**

**Search String:** ((“Randomise control trial” OR “RCT” OR “Non-randomised control trial” OR “nRCT” OR “Cohort study” OR “Retrospective study” OR “Prospective study” OR “Case series”) AND (“Efficacy” OR “Effectivenes” OR “Effectivity” OR “Safety”) AND (“Oseltamivir” OR “Tamiflu”) AND (“Treatment” OR “Management” OR “Therapy” OR “Cure”) AND (“2019 novel Coronavirus” OR “2019-nCoV” OR “Coronavirus disease 2019” OR “COVID-19” OR “Wuhan coronavirus” OR “Severe acute respiratory syndrome coronavirus 2” OR “SARS-CoV-2”))

**# Hits:**  32 (hits for the first search).

**Hit result of the repeat search:** 35 (searched on 17/7/2021).

**Library/Database:** ProQuest

**Date of Search:** March 12, 2021 **(first search date)**

**Search String:**  ((“Randomise control trial” OR “RCT” OR “Non-randomised control trial” OR “nRCT” OR “Cohort study” OR “Retrospective study” OR “Prospective study” OR “Case series”) AND (“Efficacy” OR “Effectivenes” OR “Effectivity” OR “Safety”) AND (“Oseltamivir” OR “Tamiflu”) AND (“Treatment” OR “Management” OR “Therapy” OR “Cure”) AND (“2019 novel Coronavirus” OR “2019-nCoV” OR “Coronavirus disease 2019” OR “COVID-19” OR “Wuhan coronavirus” OR “Severe acute respiratory syndrome coronavirus 2” OR “SARS-CoV-2”))

**# Hits: 365** (hits for the first search).

**Hit result of the repeat search: 371** (searched on 17/7/2021).

**Library/Database:** Embase (Via OVID)

**Date of Search:** March 12, 2021 **(first search date)**

**Search String:** All fields ((“Randomise control trial” OR “RCT” OR “Non-randomised control trial” OR “nRCT” OR “Cohort study” OR “Retrospective study” OR “Prospective study” OR “Case series”) AND (“Efficacy” OR “Effectivenes” OR “Effectivity” OR “Safety”) AND (“Oseltamivir” OR “Tamiflu”) AND (“Treatment” OR “Management” OR “Therapy” OR “Cure”) AND (“2019 novel Coronavirus” OR “2019-nCoV” OR “Coronavirus disease 2019” OR “COVID-19” OR “Wuhan coronavirus” OR “Severe acute respiratory syndrome coronavirus 2” OR “SARS-CoV-2”))

**# Hits:**  6521 (hits for the first search).

**Hit result of the repeat search: 6521** (searched on 17/7/2021).

Total # of citations prior to de-duplication: 7182

Total # of citations after 1^st^ round of de-duplication in Rayyan: 6811

Total # citations added from manual search: 2

Total # citations (de-duplicated + manual search): 6813

**Data management**

The citations obtained from the databases’ search (search results) will be uploaded (exported) to Rayan web-based software. All the screening steps of the systematic review will be carried out on Rayan. While the data extraction will be done MS Excel spreadsheet.

**Selection process**

The search and screening process of the study will be conducted by two independent reviewers and a third reviewer will decide about uncertainties.

**Data collection process**

Extraction of data will be conducted after the full text searching. The relevant information will be extracted from each article included and recorded immediately in the data extraction file. This will be carried out by two independent reviewers and two others will check the information.

**Intervention:**

Oseltamivir: alone or in combination.

**Comparisons:**

Usual care (supportive therapy), Other drugs (alone or in combination), or Placebo.

**Outcomes:**

Patient recovery from COVID-19 (survival).

**Other outcomes:**

Clinical response, virological response, laboratory response, radiological response, adverse reaction, and duration of hospitalisation.

**Risk of Bias (quality) assessment**

Only relevant articles that meets the inclusion and exclusion criteria of the study will be included. The quality of the included articles will be further evaluated based on the; 1) Cochrane risk of bias tool (RoB 1.0) for randomized controlled studies [[4](#_ENREF_4)], 2) the Newcastle Ottawa Scale for observational studies, for non-randomized interventional studies [[6](#_ENREF_6)]. The appropriate critical appraisal checklist will be applied for each study design. Two independent reviewers will conduct the critical appraisal and a third reviewer will cross-check the appraisal process.

**Meta-analysis**

**Statistical Assessment**

RevMan5.3 software (Nordic Cochran Centre, Copenhagen, Denmark) provided by Cochrane Collaboration is used for quantitative synthesis and analysis of data. For continuous variable, data will be pooled using standard mean difference. While for dichotomous data, either odd ratio (OR), risk ratio (RR) together with a 95% confidence interval (CI) will be used. Analysis will be done using the random effect model. If there is not enough article for meta-analysis or the articles are of poor quality meta-analysis will not be done and only descriptive analysis will be done.

**Assessment of Heterogeneity**

Statistical heterogeneity among the studies included will be estimated using the ***X^2^*** test and ***I^2^*** statistics. An ***I^2^*** value of 0 to <40% will not be considered significant, 30% to 60% will be regarded as moderate heterogeneity, 50% to 90% will be considered substantial heterogeneity, and 75% to 100% will be considered significant heterogeneity.

**Sensitivity Analysis**

Sensitivity analysis will be done based on leave one out model to identify the studies that greatly influence the result of the meta-analysis.

**Subgroup Analysis**

Subgroup analysis will be considered, if necessary, based on the distribution of the included studies for meta-analysis.

**Publication bias**

If the number of studies to be included in the meta-analysis is ≥10, the funnel plot will be used to exam for publication bias, and the Egger test will be used to evaluate the symmetry of the funnel plot.

**References**

1. Kimberlin, D.W., *295 - Antiviral Agents*, in *Principles and Practice of Pediatric Infectious Diseases (Fifth Edition)*, S.S. Long, C.G. Prober, and M. Fischer, Editors. 2018, Elsevier. p. 1551-1567.e6.

2. Ison, M.G. and F.G. Hayden, *154 - Antiviral Agents Against Respiratory Viruses*, in *Infectious Diseases (Fourth Edition)*, J. Cohen, W.G. Powderly, and S.M. Opal, Editors. 2017, Elsevier. p. 1318-1326.e2.

3. Zhang, X.W. and Y.L. Yap, *The 3D structure analysis of SARS-CoV S1 protein reveals a link to influenza virus neuraminidase and implications for drug and antibody discovery.* Journal of Molecular Structure: THEOCHEM, 2004. **681**(1-3): p. 137-141.

4. Sterne, J.A., et al., *RoB 2: a revised tool for assessing risk of bias in randomised trials.* bmj, 2019. **366**.

5. Sterne, J.A., et al., *ROBINS-I: a tool for assessing risk of bias in non-randomised studies of interventions.* bmj, 2016. **355**.

6. Wells, G.A., et al., *The Newcastle-Ottawa Scale (NOS) for assessing the quality of nonrandomised studies in meta-analyses*. 2000, Oxford.
