# Supplementary material for: Systematic review and meta-analysis of the efficacy and safety of oseltamivir (Tamiflu) in the treatment of Coronavirus Disease 2019 (COVID-19)
Source: PLoS One. 2022 Dec 1;17(12):e0277206. doi: 10.1371/journal.pone.0277206 (PMC9714710; doi:10.1371/journal.pone.0277206)
Supplement: S4 File — (DOCX) [file pone.0277206.s004.docx]

**S4 File: NEWCASTLE-OTTAWA QUALITY ASSESSMENT SCALE**

**NEWCASTLE - OTTAWA QUALITY ASSESSMENT SCALE**

**CASE CONTROL STUDIES**

Note: A study can be awarded a maximum of one star for each numbered item within the Selection and Exposure categories. A maximum of two stars can be given for Comparability.

**Selection:**

1) Is the case definition adequate?

a) yes, with independent validation **¯**

b) yes, eg record linkage or based on self reports

c) no description

2) Representativeness of the cases

a) consecutive or obviously representative series of cases **¯**

b) potential for selection biases or not stated

3) Selection of Controls

a) community controls **¯**

b) hospital controls

c) no description

4) Definition of Controls

a) no history of disease (endpoint) **¯**

b) no description of source

**Comparability:**

1) Comparability of cases and controls on the basis of the design or analysis

a) study controls for _______________ (Select the most important factor.) **¯**

b) study controls for any additional factor **¯** (This criteria could be modified to indicate specific

control for a second important factor.)

**Exposure:**

1) Ascertainment of exposure

a) secure record (eg surgical records) **¯**

b) structured interview where blind to case/control status **¯**

c) interview not blinded to case/control status

d) written self report or medical record only

e) no description

2) Same method of ascertainment for cases and controls

a) yes **¯**

b) no

3) Non-Response rate

a) same rate for both groups **¯**

b) non respondents described

c) rate different and no designation

**NEWCASTLE - OTTAWA QUALITY ASSESSMENT SCALE**

**COHORT STUDIES**

Note: A study can be awarded a maximum of one star for each numbered item within the Selection and Outcome categories. A maximum of two stars can be given for Comparability.

**Selection:**

1) Representativeness of the exposed cohort

a) truly representative of the average _______________ (describe) in the community **¯**

b) somewhat representative of the average ______________ in the community **¯**

c) selected group of users eg nurses, volunteers

d) no description of the derivation of the cohort

2) Selection of the non exposed cohort

a) drawn from the same community as the exposed cohort **¯**

b) drawn from a different source

c) no description of the derivation of the non exposed cohort

3) Ascertainment of exposure

a) secure record (eg surgical records) **¯**

b) structured interview **¯**

c) written self report

d) no description

4) Demonstration that outcome of interest was not present at start of study

a) yes **¯**

b) no

**Comparability:**

1) Comparability of cohorts on the basis of the design or analysis

a) study controls for _____________ (select the most important factor) **¯**

b) study controls for any additional factor **¯** (This criteria could be modified to indicate specific

control for a second important factor.)

**Outcome:**

1) Assessment of outcome

a) independent blind assessment **¯**

b) record linkage **¯**

c) self report

d) no description

2) Was follow-up long enough for outcomes to occur

a) yes (select an adequate follow up period for outcome of interest) **¯**

b) no

3) Adequacy of follow up of cohorts

a) complete follow up - all subjects accounted for **¯**

b) subjects lost to follow up unlikely to introduce bias - small number lost - > ____ % (select an

adequate %) follow up, or description provided of those lost) **¯**

c) follow up rate < ____% (select an adequate %) and no description of those lost

d) no statement

KEY: Thresholds for converting the Newcastle-Ottawa scales to AHRQ standards (good, fair, and poor):

**Good quality:** 3 or 4 stars in selection domain AND 1 or 2 stars in comparability domain AND 2 or 3 stars in outcome/exposure domain.

**Fair quality:** 2 stars in selection domain AND 1 or 2 stars in comparability domain AND 2 or 3 stars in outcome/exposure domain.

**Poor quality:** 0 or 1 star in selection domain OR 0 stars in comparability domain OR 0 or 1 stars in outcome/exposure domain.

**NEWCASTLE -OTTAWA QUALITY ASSESSMENT SCALE FOR CROSS-SECTIONAL STUDIES**

**Selection:**

**1.Representativeness of the sample:**

- 1. a. Truly representative of the average in the target population. * (all subjects or random sampling)
  2. b. Somewhat representative of the average in the target group. * (non-random sampling)
  3. c. Selected group of users/convenience sample.
  4. d. No description of the derivation of the included subjects.
  5. **2. Sample size:**
  6. a. Justified and satisfactory (including sample size calculation). *
  7. b. Not justified.
  8. c. No information provided.
  9. 3. Non-respondents:
  10. a. Proportion of target sample recruited attains pre-specified target or basic summary of non-respondent characteristics in sampling frame recorded. *
  11. b. Unsatisfactory recruitment rate, no summary data on non-respondents.
  12. c. No information provided.

4. Ascertainment of the exposure (risk factor):

- 1. a. Vaccine records/vaccine registry/clinic registers/hospital records only. **
  2. b. Parental or personal recall and vaccine/hospital records. *
  3. c. Parental/personal recall only.

**Comparability:** (Maximum 2 stars)

- 1. 1. Comparability of subjects in different outcome groups on the basis of design or analysis. Confounding factors controlled.
  2. a. Data/ results adjusted for relevant predictors/risk factors/confounders e.g. age, sex, time since vaccination, etc. **
  3. b. Data/results not adjusted for all relevant confounders/risk factors/information not provided.

**Outcome:**

- 1. 1. Assessment of outcome:
  2. a. Independent blind assessment using objective validated laboratory methods. **
  3. b. Unblinded assessment using objective validated laboratory methods. **
  4. c. Used non-standard or non-validated laboratory methods with gold standard. *
  5. d. No description/non-standard laboratory methods used.
  6. 2. Statistical test:
  7. a. Statistical test used to analyse the data clearly described, appropriate and measures of association presented including confidence intervals and probability level (p value). *
  8. b. Statistical test not appropriate, not described or incomplete.

KEY: Interpretation of Cross-sectional Studies:

Very Good Studies: 9-10 points

Good Studies: 7-8 points

Satisfactory Studies: 5-6 points

Unsatisfactory Studies: 0 to 4 points
