# Supplementary material for: Systematic review and meta-analysis of the efficacy and safety of oseltamivir (Tamiflu) in the treatment of Coronavirus Disease 2019 (COVID-19)
Source: PLoS One. 2022 Dec 1;17(12):e0277206. doi: 10.1371/journal.pone.0277206 (PMC9714710; doi:10.1371/journal.pone.0277206)

S5 File

Conducting TSA

For the primary outcome, the Peto’s odds ratio (being the preferred effect measure for meta-analysis of rare events) was used as the effect measure for the meta-analysis on the TSA programme. The Sidik-Jonkman (SJ) random effect model was used and the conventional (coverage) approach was adopted for the estimation of the adjusted significance test boundaries at a 95% confidence interval (CI). To calculate the required information size (RIS), (accumulated) sample size was selected as the type of information. The O’Brien-Fleming α-spending function was used to adjust for a two-sided z-score threshold with 80% power, and 2-sided 5% type 1 error. The relative risk reduction (RRR) rate was estimated to be 35% and the event rate in the control group was estimated as 3%. While the heterogeneity correction was estimated at 45%. Estimate values for RRR above or below 35% could not be used due to too low information use (<1.0% and 4.29% respectively). Likewise for control group event rate, and heterogeneity correction, values above or below the provided estimates could not be used due to too little information use. However, to ensure robustness and account for the observed high heterogeneity, the TSA was repeated by removing one study for sensitivity analysis.

As for the duration of hospitalisation outcome, the TSA was calculated using mean difference as the effect measure. Also, the SJ random effect model and the conventional approach for the estimation of the adjusted significance test boundaries at 95% CI were used. The parameters used in the calculation of the RIS were the same as that of the primary outcome. However, the low bias estimation was used for the determination of variance and mean difference. While for the estimation of heterogeneity correction the model variance-based was used.


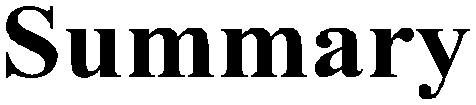


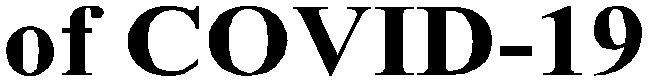


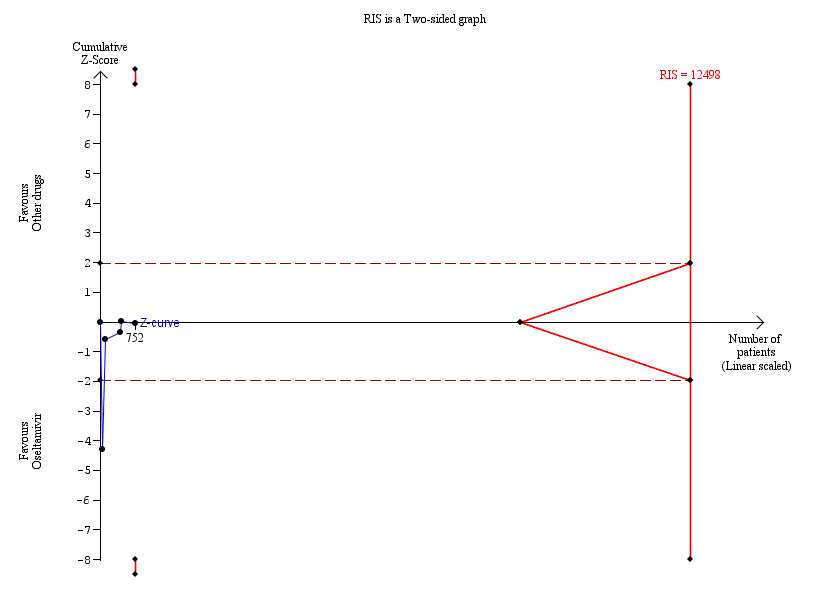


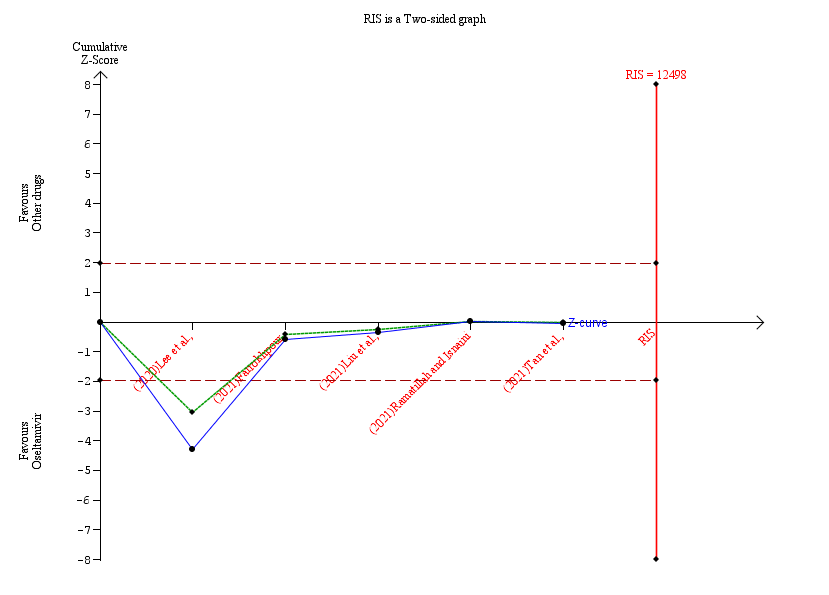


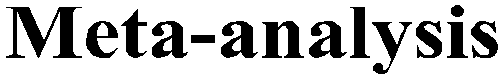


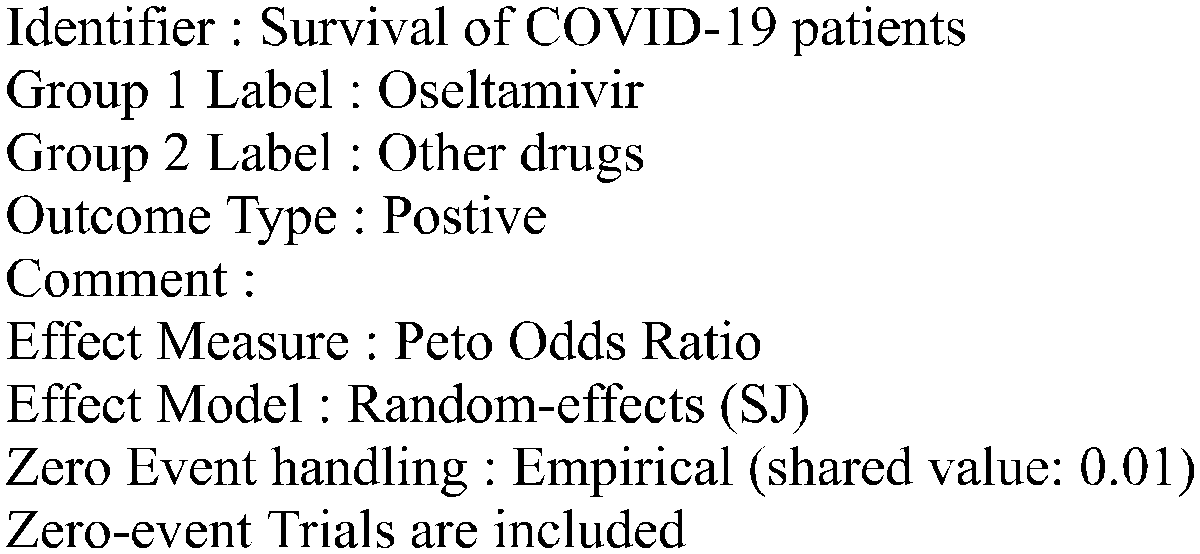


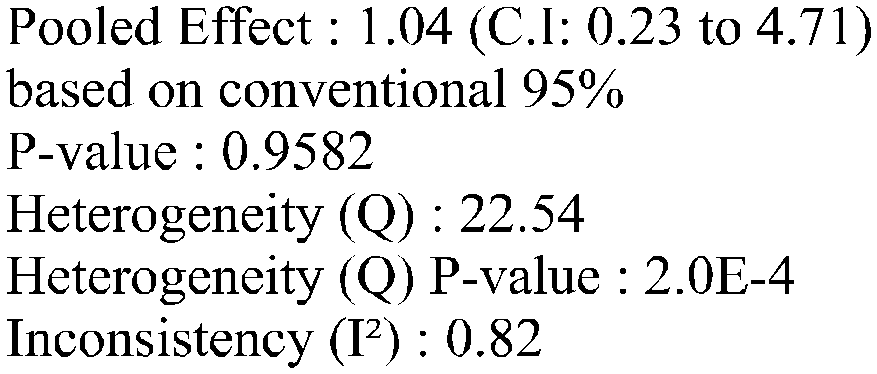


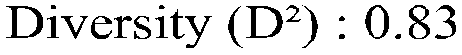


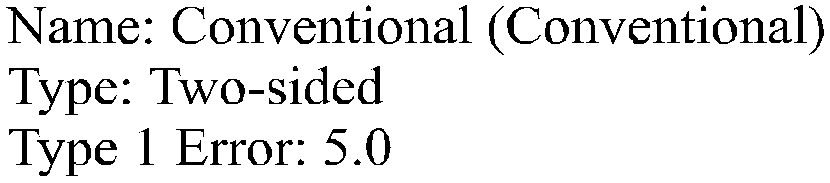


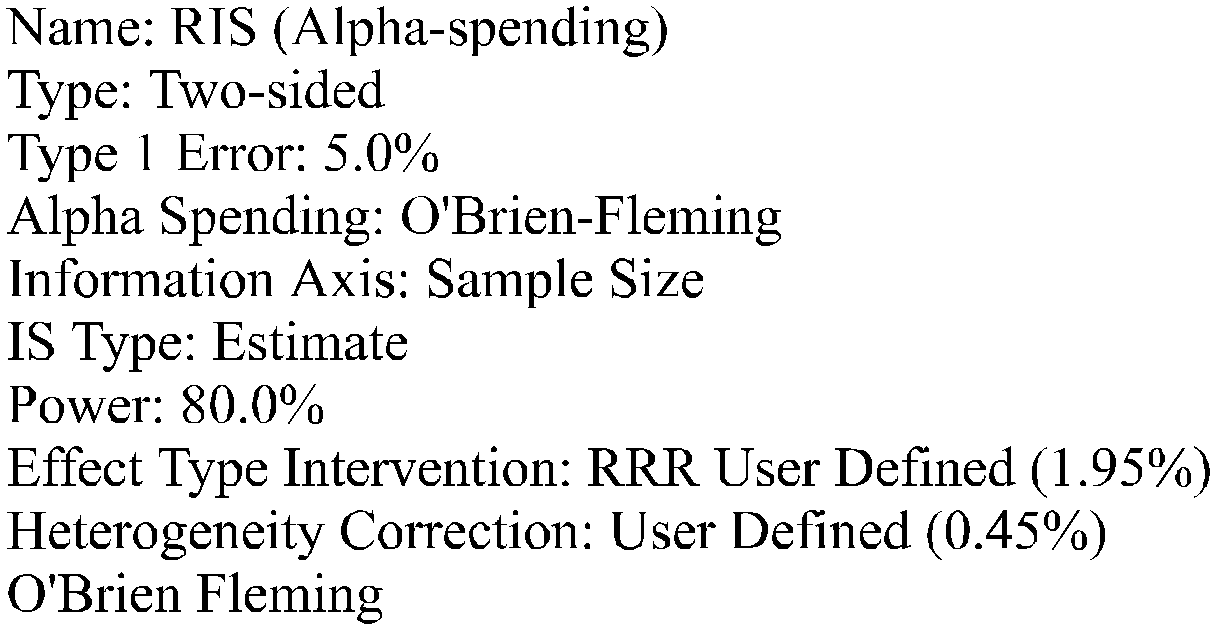


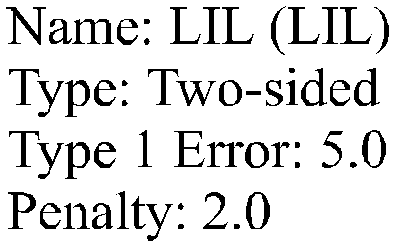


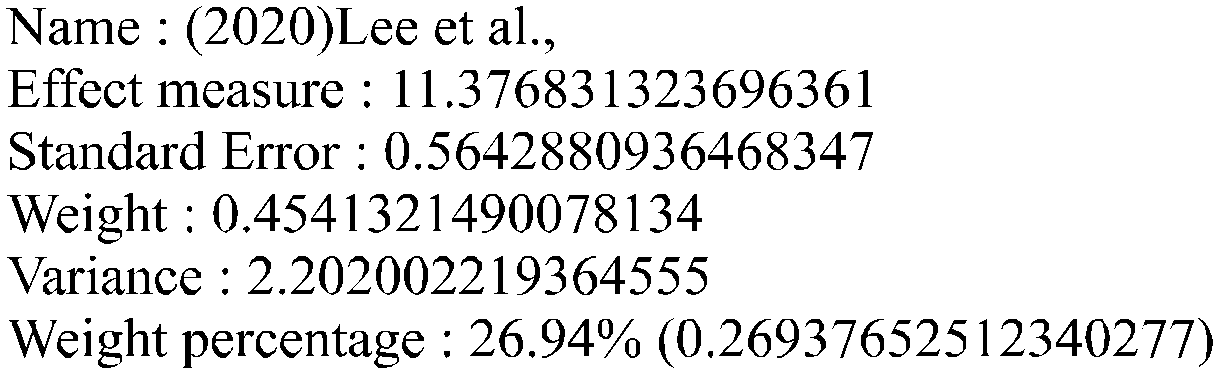


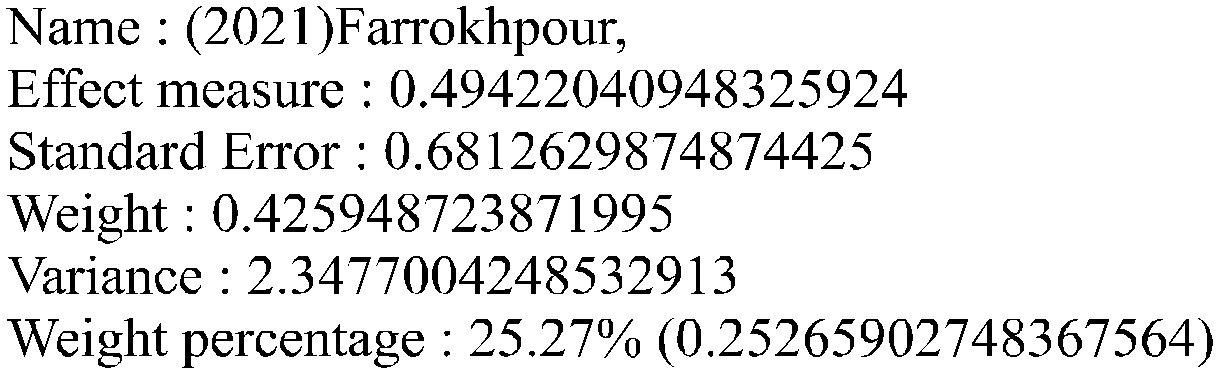


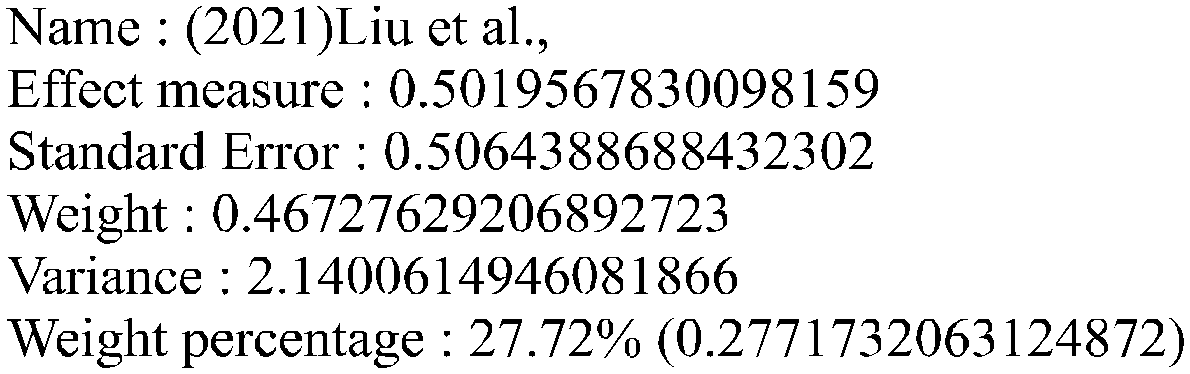


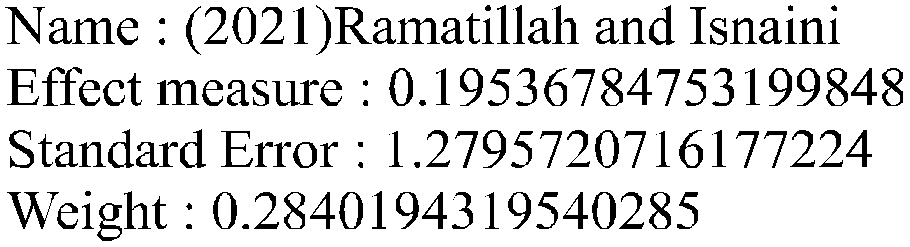


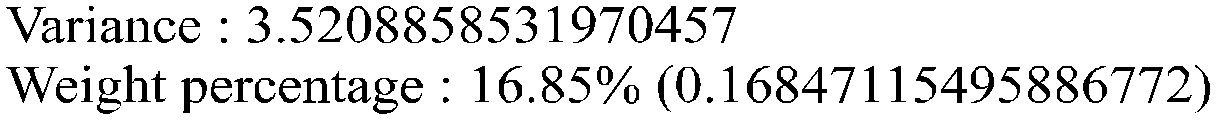


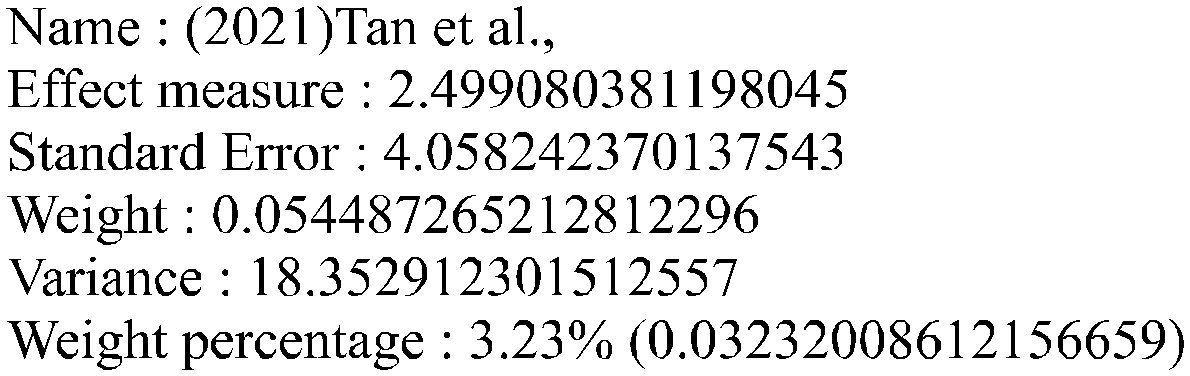

Supplement: S5 File — (DOC) [file pone.0277206.s005.doc]
