# Supplementary material for: Systematic review and meta-analysis of the efficacy and safety of oseltamivir (Tamiflu) in the treatment of Coronavirus Disease 2019 (COVID-19)
Source: PLoS One. 2022 Dec 1;17(12):e0277206. doi: 10.1371/journal.pone.0277206 (PMC9714710; doi:10.1371/journal.pone.0277206)
Supplement: S6 File — (DOCX) [file pone.0277206.s006.docx]

**S6 File**

**ROBINS-I tool (Stage I):**

Review question: What is the efficacy and safety of Oseltamivir (Tamiflu) alone or in combination with any drug in the treatment of patients diagnosed of COVID-19 using published literature.

|  | Participants | all patients that meet the diagnostic criteria of COVID-19 according to the standard guideline stipulated or adopted in the country of the research. |
| --- | --- | --- |
|  | Intervention | Oseltamivir alone or in combination |
|  | Comparator | Usual care, other drugs (alone or in combination), Placebo |
|  | Outcome(s) | Patient recovery from COVID-19 (survival), Clinical response, virological response, laboratory response, radiological response. |

List the confounding domains relevant to all or most studies

| Age, Sex, Comorbidity, Co-infection, Co-intervention, hospital/location, |
| --- |

List co-interventions that could be different between intervention groups and that could impact on outcomes

| Antibiotics, Antihypertension, Antidiabetic, Oxygen therapy, |
| --- |

**ROBINS-I tool (Stage II):**

**S**pecify a target randomized trial specific to the study:

Evaluation of The Efficacy of Oseltamivir versus Arbidol therapy in Patients Infected With COVID-19

|  | Design | Randomized control trial |
| --- | --- | --- |
|  | Participants | Patients Infected With COVID-19 |
|  | Intervention | Standard treatment with oseltamivir |
|  | comparators | Arbidol |

The aim for this study:

To assess the effect of starting and adhering to intervention

Specify the outcome(s):

| Patient recovery from COVID-19 (survival), Clinical response, virological response, laboratory response, radiological response, adverse effects, and duration of hospitalisation. |
| --- |

Specify the numerical result being assessed:

| Odds ratio (OR), Mean difference (MD) |
| --- |

Risk of bias assessment for the eight included studies

Domain 1: Bias due to confounding

|  | Relevant signalling questions | Response | Elaboration/remarks |
| --- | --- | --- | --- |
| Lee et al., 2020 | 1.1 Is there potential for confounding of the effect of intervention in this study? | Y | Confounding such as those due to comorbidities, medications, and disease severity etc. |
|  | Determine whether there is need to assess time-varying confounding | N |  |
|  | 1.2 Was the analysis based on splitting the participants’ follow-up time according to the intervention received? | N |  |
|  | 1.4 Did the authors use an appropriate analysis method that controlled for all the important confounding domains? | Y | Authors used two models of multivariate logistic regression analysis. |
|  | 1.5 Were confounding domains that were controlled for measured validly and reliably by the variables available in this study? | Y |  |
|  | Did the authors control for any post-intervention variables that could have been affected by the intervention? | N |  |
| Response for confounding domain | Moderate risk of bias | - | Confounding is expected but all known important confounding domains were appropriately measured and controlled for; and reliability and validity of important domains were sufficient, such that we do not expect serious residual confounding. |

Domain 2: Bias in selection of participants into the study

|  | Relevant signalling questions | Response | Elaboration/remarks |
| --- | --- | --- | --- |
| Lee et al., 2020 | 2.1 Was selection of participants into the study (or into the analysis) based on participant characteristics observed after the start of intervention? | PN |  |
|  | 2.4 Do start of follow-up and start of intervention coincide for most participants? | PY |  |
| Response for domain 2 | Low risk of bias | - | All eligible participants were included in the study; and each participants’ start of follow-up and start of intervention coincided. |

Domain 3: Bias in classification of intervention

|  | Relevant signalling questions | Response | Elaboration/remarks |
| --- | --- | --- | --- |
| Lee et al., 2020 | 3.1 Were intervention groups clearly defined? | Y |  |
|  | 3.2 Was the information used to define the intervention groups recorded at the start of the intervention? | Y |  |
|  | 3.3 Could classification of intervention status have been affected by knowledge of the outcome or risk of the outcome? | PY |  |
| Response to domain 3 | Low risk of bias | - | Intervention status is well defined and intervention definition is based solely on information collected at the time of intervention. |

Domain 4: Bias due to deviation from intended intervention

|  | Relevant signalling questions | Response | Elaboration/remarks |
| --- | --- | --- | --- |
| Lee et al., 2020 | 4.3 Were important co-interventions balanced across intervention groups? | PY |  |
|  | 4.4 Was the intervention implemented successfully for most participants? | PY |  |
|  | 4.5 Did study participants adhere to the assigned intervention regimen? | PY |  |
| Response to domain 4 | Low risk of bias | - | The important co-interventions were balanced across intervention groups and there were no deviations from the intended interventions (in terms of implementation of adherence) that were likely to impact on the outcome. |

Domain 5: Bias due to missing data

|  | Relevant signalling questions | Response | Elaboration/remarks |
| --- | --- | --- | --- |
| Lee et al., 2020 | 5.1 Were outcome data available for all, or nearly all, participants? | Y |  |
|  | 5.2 Were participants excluded due to missing data on intervention status? | PY |  |
|  | 5.3 Were participants excluded due to missing data on other variables needed for the analysis? | PY |  |
|  | 5.4 Are proportion of participants and reasons for missing data similar across interventions? | NI |  |
| Response to domain 5 | Low risk of bias | - | Data were reasonably complete. |

Domain 6: Bias in measurement of outcome

|  | Relevant signalling questions | Response | Elaboration/remarks |
| --- | --- | --- | --- |
| Lee et al., 2020 | 6.1 Could the outcome measured have been influenced by knowledge of the intervention received? | Y |  |
|  | 6.2 Were outcome assessors aware of the intervention received by the study participants? | PY |  |
|  | 6.3 Were the methods of outcome assessment comparable across intervention groups? | Y |  |
|  | 6.4 Were any systematic errors in measurement of the outcomes related to intervention received? | NI |  |
| Response to domain 6 | Low risk of bias | - | The methods of outcome assessment were comparable across intervention groups. |

Domain 7: Bias in selection of the reported results

|  | Relevant signalling questions | Response | Elaboration/remarks |
| --- | --- | --- | --- |
| Lee et al., 2020 | 7.1 Is the reported effect estimate likely to be selected, on the basis of the results, from multiple outcome measurements within the outcome domain? | Y |  |
|  | 7.2 Is the reported effect estimate likely to be selected, on the basis of the results, from multiple analyses of the intervention outcome relationship? | PY |  |
|  | 7.3 Is the reported effect estimate likely to be selected, on the basis of the results, from different subroups? | Y |  |
| Response to domain 7 | moderate risk of bias | - | The outcome measurement and analyses are consistent with an a *priori* plan. |

Domain 1: Bias due to confounding

|  | Relevant signalling questions | Response | Elaboration/remarks |
| --- | --- | --- | --- |
| Liu et al., 2021 | 1.1 Is there potential for confounding of the effect of intervention in this study? | Y | Confounding such as those due to sex, pre-existing condition, medication use, hospital, age, SpO2 level, and admission date. |
|  | Determine whether there is need to assess time-varying confounding | N |  |
|  | 1.2 Was the analysis based on splitting the participants’ follow-up time according to the intervention received? | N |  |
|  | 1.4 Did the authors use an appropriate analysis method that controlled for all the important confounding domains? | Y | Authors used logistic regression analysis. |
|  | 1.5 Were confounding domains that were controlled for measured validly and reliably by the variables available in this study? | Y |  |
|  | Did the authors control for any post-intervention variables that could have been affected by the intervention? | N |  |
| Response for confounding domain | Moderate risk of bias | - | Confounding is expected but all known important confounding domains were appropriately measured and controlled for; and reliability and validity of important domains were sufficient, such that we do not expect serious residual confounding. |

Domain 2: Bias in selection of participants into the study

|  | Relevant signalling questions | Response | Elaboration/remarks |
| --- | --- | --- | --- |
| Liu et al., 2021 | 2.1 Was selection of participants into the study (or into the analysis) based on participant characteristics observed after the start of intervention? | N |  |
|  | 2.4 Do start of follow-up and start of intervention coincide for most participants? | PY |  |
| Response for domain 2 | Low risk of bias | - | All eligible participants were included in the study; and each participants’ start of follow-up and start of intervention coincided. |

Domain 3: Bias in classification of intervention

|  | Relevant signalling questions | Response | Elaboration/remarks |
| --- | --- | --- | --- |
| Liu et al., 2021 | 3.1 Were intervention groups clearly defined? | Y |  |
|  | 3.2 Was the information used to define the intervention groups recorded at the start of the intervention? | Y |  |
|  | 3.3 Could classification of intervention status have been affected by knowledge of the outcome or risk of the outcome? | PY |  |
| Response to domain 3 | Low risk of bias | - | Intervention status is well defined and intervention definition is based solely on information collected at the time of intervention. |

Domain 4: Bias due to deviation from intended intervention

|  | Relevant signalling questions | Response | Elaboration/remarks |
| --- | --- | --- | --- |
| Liu et al., 2021 | 4.3 Were important co-interventions balanced across intervention groups? | Y |  |
|  | 4.4 Was the intervention implemented successfully for most participants? | Y |  |
|  | 4.5 Did study participants adhere to the assigned intervention regimen? | PY |  |
| Response to domain 4 | Low risk of bias | - | The important co-interventions were balanced across intervention groups and there were no deviations from the intended interventions (in terms of implementation of adherence) that were likely to impact on the outcome. |

Domain 5: Bias due to missing data

|  | Relevant signalling questions | Response | Elaboration/remarks |
| --- | --- | --- | --- |
| Liu et al., 2021 | 5.1 Were outcome data available for all, or nearly all, participants? | Y |  |
|  | 5.2 Were participants excluded due to missing data on intervention status? | Y |  |
|  | 5.3 Were participants excluded due to missing data on other variables needed for the analysis? | PY |  |
|  | 5.4 Are proportion of participants and reasons for missing data similar across interventions? | NI |  |
| Response to domain 5 | Low risk of bias | - | Data were reasonably complete. |

Domain 6: Bias in measurement of outcome

|  | Relevant signalling questions | Response | Elaboration/remarks |
| --- | --- | --- | --- |
| Liu et al., 2021 | 6.1 Could the outcome measured have been influenced by knowledge of the intervention received? | Y |  |
|  | 6.2 Were outcome assessors aware of the intervention received by the study participants? | PY |  |
|  | 6.3 Were the methods of outcome assessment comparable across intervention groups? | Y |  |
|  | 6.4 Were any systematic errors in measurement of the outcomes related to intervention received? | NI |  |
| Response to domain 6 | Low risk of bias | - | The methods of outcome assessment were comparable across intervention groups. |

Domain 7: Bias in selection of the reported results

|  | Relevant signalling questions | Response | Elaboration/remarks |
| --- | --- | --- | --- |
| Liu et al., 2021 | 7.1 Is the reported effect estimate likely to be selected, on the basis of the results, from multiple outcome measurements within the outcome domain? | Y |  |
|  | 7.2 Is the reported effect estimate likely to be selected, on the basis of the results, from multiple analyses of the intervention outcome relationship? | PY |  |
|  | 7.3 Is the reported effect estimate likely to be selected, on the basis of the results, from different subroups? | Y |  |
| Response to domain 7 | moderate risk of bias | - | The outcome measurement and analyses are consistent with an a *priori* plan. |

Domain 1: Bias due to confounding

|  | Relevant signalling questions | Response | Elaboration/remarks |
| --- | --- | --- | --- |
| Tan et al., 2021 | 1.1 Is there potential for confounding of the effect of intervention in this study? | Y | Confounding such as those due to sex, pre-existing condition, medication use, hospital, age, SpO2 level, and admission date. |
|  | Determine whether there is need to assess time-varying confounding | N |  |
|  | 1.2 Was the analysis based on splitting the participants’ follow-up time according to the intervention received? | N |  |
|  | 1.4 Did the authors use an appropriate analysis method that controlled for all the important confounding domains? | N |  |
|  | 1.5 Were confounding domains that were controlled for measured validly and reliably by the variables available in this study? | NI |  |
|  | Did the authors control for any post-intervention variables that could have been affected by the intervention? | N |  |
| Response for confounding domain | Serious risk of bias | - | At least one known important domain was not appropriately measured and controlled for. |

Domain 2: Bias in selection of participants into the study

|  | Relevant signalling questions | Response | Elaboration/remarks |
| --- | --- | --- | --- |
| Tan et al., 2021 | 2.1 Was selection of participants into the study (or into the analysis) based on participant characteristics observed after the start of intervention? | N |  |
|  | 2.4 Do start of follow-up and start of intervention coincide for most participants? | Y |  |
| Response for domain 2 | Low risk of bias | - | All eligible participants were included in the study; and each participants’ start of follow-up and start of intervention coincided. |

Domain 3: Bias in classification of intervention

|  | Relevant signalling questions | Response | Elaboration/remarks |
| --- | --- | --- | --- |
| Tan et al., 2021 | 3.1 Were intervention groups clearly defined? | Y |  |
|  | 3.2 Was the information used to define the intervention groups recorded at the start of the intervention? | Y |  |
|  | 3.3 Could classification of intervention status have been affected by knowledge of the outcome or risk of the outcome? | PY |  |
| Response to domain 3 | Low risk of bias | - | Intervention status is well defined and intervention definition is based solely on information collected at the time of intervention. |

Domain 4: Bias due to deviation from intended intervention

|  | Relevant signalling questions | Response | Elaboration/remarks |
| --- | --- | --- | --- |
| Tan et al., 2021 | 4.3 Were important co-interventions balanced across intervention groups? | NI |  |
|  | 4.4 Was the intervention implemented successfully for most participants? | Y |  |
|  | 4.5 Did study participants adhere to the assigned intervention regimen? | PY |  |
| Response to domain 4 | Moderate risk of bias | - | The important co-interventions were not balanced across intervention groups. |

Domain 5: Bias due to missing data

|  | Relevant signalling questions | Response | Elaboration/remarks |
| --- | --- | --- | --- |
| Tan et al., 2021 | 5.1 Were outcome data available for all, or nearly all, participants? | Y |  |
|  | 5.2 Were participants excluded due to missing data on intervention status? | NI |  |
|  | 5.3 Were participants excluded due to missing data on other variables needed for the analysis? | PN |  |
|  | 5.4 Are proportion of participants and reasons for missing data similar across interventions? | NI |  |
| Response to domain 5 | Low risk of bias | - | Data were reasonably complete. |

Domain 6: Bias in measurement of outcome

|  | Relevant signalling questions | Response | Elaboration/remarks |
| --- | --- | --- | --- |
| Tan et al., 2021 | 6.1 Could the outcome measured have been influenced by knowledge of the intervention received? | Y |  |
|  | 6.2 Were outcome assessors aware of the intervention received by the study participants? | PY |  |
|  | 6.3 Were the methods of outcome assessment comparable across intervention groups? | Y |  |
|  | 6.4 Were any systematic errors in measurement of the outcomes related to intervention received? | NI |  |
| Response to domain 6 | Low risk of bias | - | The methods of outcome assessment were comparable across intervention groups. |

Domain 7: Bias in selection of the reported results

|  | Relevant signalling questions | Response | Elaboration/remarks |
| --- | --- | --- | --- |
| Tan et al., 2021 | 7.1 Is the reported effect estimate likely to be selected, on the basis of the results, from multiple outcome measurements within the outcome domain? | Y |  |
|  | 7.2 Is the reported effect estimate likely to be selected, on the basis of the results, from multiple analyses of the intervention outcome relationship? | PY |  |
|  | 7.3 Is the reported effect estimate likely to be selected, on the basis of the results, from different subroups? | Y |  |
| Response to domain 7 | moderate risk of bias | - | The outcome measurement and analyses are consistent with an a *priori* plan. |

Domain 1: Bias due to confounding

|  | Relevant signalling questions | Response | Elaboration/remarks |
| --- | --- | --- | --- |
| Farrokhpour et al, 2021 | 1.1 Is there potential for confounding of the effect of intervention in this study? | Y | Confounding such as those due to age, sex, and pre-existing condition. |
|  | Determine whether there is need to assess time-varying confounding | N |  |
|  | 1.2 Was the analysis based on splitting the participants’ follow-up time according to the intervention received? | N |  |
|  | 1.4 Did the authors use an appropriate analysis method that controlled for all the important confounding domains? | Y | Authors used regression analysis. |
|  | 1.5 Were confounding domains that were controlled for measured validly and reliably by the variables available in this study? | Y |  |
|  | Did the authors control for any post-intervention variables that could have been affected by the intervention? | N |  |
| Response for confounding domain | Moderate risk of bias | - | Confounding is expected but all known important confounding domains were appropriately measured and controlled for; and reliability and validity of important domains were sufficient, such that we do not expect serious residual confounding. |

Domain 2: Bias in selection of participants into the study

|  | Relevant signalling questions | Response | Elaboration/remarks |
| --- | --- | --- | --- |
| Farrokhpour et al, 2021 | 2.1 Was selection of participants into the study (or into the analysis) based on participant characteristics observed after the start of intervention? | N |  |
|  | 2.4 Do start of follow-up and start of intervention coincide for most participants? | PY |  |
| Response for domain 2 | Low risk of bias | - | All eligible participants were included in the study; and each participants’ start of follow-up and start of intervention coincided. |

Domain 3: Bias in classification of intervention

|  | Relevant signalling questions | Response | Elaboration/remarks |
| --- | --- | --- | --- |
| Farrokhpour et al, 2021 | 3.1 Were intervention groups clearly defined? | Y |  |
|  | 3.2 Was the information used to define the intervention groups recorded at the start of the intervention? | Y |  |
|  | 3.3 Could classification of intervention status have been affected by knowledge of the outcome or risk of the outcome? | PY |  |
| Response to domain 3 | Low risk of bias | - | Intervention status is well defined and intervention definition is based solely on information collected at the time of intervention. |

Domain 4: Bias due to deviation from intended intervention

|  | Relevant signalling questions | Response | Elaboration/remarks |
| --- | --- | --- | --- |
| Farrokhpour et al, 2021 | 4.3 Were important co-interventions balanced across intervention groups? | Y |  |
|  | 4.4 Was the intervention implemented successfully for most participants? | Y |  |
|  | 4.5 Did study participants adhere to the assigned intervention regimen? | PY |  |
| Response to domain 4 | Low risk of bias | - | The important co-interventions were balanced across intervention groups and there were no deviations from the intended interventions (in terms of implementation of adherence) that were likely to impact on the outcome. |

Domain 5: Bias due to missing data

|  | Relevant signalling questions | Response | Elaboration/remarks |
| --- | --- | --- | --- |
| Farrokhpour et al, 2021 | 5.1 Were outcome data available for all, or nearly all, participants? | Y |  |
|  | 5.2 Were participants excluded due to missing data on intervention status? | Y |  |
|  | 5.3 Were participants excluded due to missing data on other variables needed for the analysis? | PY |  |
|  | 5.4 Are proportion of participants and reasons for missing data similar across interventions? | NI |  |
| Response to domain 5 | Low risk of bias | - | Data were reasonably complete. |

Domain 6: Bias in measurement of outcome

|  | Relevant signalling questions | Response | Elaboration/remarks |
| --- | --- | --- | --- |
| Farrokhpour et al, 2021 | 6.1 Could the outcome measured have been influenced by knowledge of the intervention received? | Y |  |
|  | 6.2 Were outcome assessors aware of the intervention received by the study participants? | PY |  |
|  | 6.3 Were the methods of outcome assessment comparable across intervention groups? | Y |  |
|  | 6.4 Were any systematic errors in measurement of the outcomes related to intervention received? | NI |  |
| Response to domain 6 | Low risk of bias | - | The methods of outcome assessment were comparable across intervention groups. |

Domain 7: Bias in selection of the reported results

|  | Relevant signalling questions | Response | Elaboration/remarks |
| --- | --- | --- | --- |
| Farrokhpour et al, 2021 | 7.1 Is the reported effect estimate likely to be selected, on the basis of the results, from multiple outcome measurements within the outcome domain? | Y |  |
|  | 7.2 Is the reported effect estimate likely to be selected, on the basis of the results, from multiple analyses of the intervention outcome relationship? | PY |  |
|  | 7.3 Is the reported effect estimate likely to be selected, on the basis of the results, from different subgroups? | Y |  |
| Response to domain 7 | moderate risk of bias | - | The outcome measurement and analyses are consistent with an a *priori* plan. |

Domain 1: Bias due to confounding

|  | Relevant signalling questions | Response | Elaboration/remarks |
| --- | --- | --- | --- |
| Rahamatillah and Isnaini, 2021 | 1.1 Is there potential for confounding of the effect of intervention in this study? | Y | Confounding such as those due to age, sex, and pre-existing condition. |
|  | Determine whether there is need to assess time-varying confounding | N |  |
|  | 1.2 Was the analysis based on splitting the participants’ follow-up time according to the intervention received? | N |  |
|  | 1.4 Did the authors use an appropriate analysis method that controlled for all the important confounding domains? | Y | Authors used stratification. |
|  | 1.5 Were confounding domains that were controlled for measured validly and reliably by the variables available in this study? | Y |  |
|  | Did the authors control for any post-intervention variables that could have been affected by the intervention? | N |  |
| Response for confounding domain | Moderate risk of bias | - | Confounding is expected but all known important confounding domains were appropriately measured and controlled for; and reliability and validity of important domains were sufficient, such that we do not expect serious residual confounding. |

Domain 2: Bias in selection of participants into the study

|  | Relevant signalling questions | Response | Elaboration/remarks |
| --- | --- | --- | --- |
| Rahamatillah and Isnaini, 2021 | 2.1 Was selection of participants into the study (or into the analysis) based on participant characteristics observed after the start of intervention? | N |  |
|  | 2.4 Do start of follow-up and start of intervention coincide for most participants? | PY |  |
| Response for domain 2 | Low risk of bias | - | All eligible participants were included in the study; and each participants’ start of follow-up and start of intervention coincided. |

Domain 3: Bias in classification of intervention

|  | Relevant signalling questions | Response | Elaboration/remarks |
| --- | --- | --- | --- |
| Rahamatillah and Isnaini, 2021 | 3.1 Were intervention groups clearly defined? | Y |  |
|  | 3.2 Was the information used to define the intervention groups recorded at the start of the intervention? | Y |  |
|  | 3.3 Could classification of intervention status have been affected by knowledge of the outcome or risk of the outcome? | PY |  |
| Response to domain 3 | Low risk of bias | - | Intervention status is well defined and intervention definition is based solely on information collected at the time of intervention. |

Domain 4: Bias due to deviation from intended intervention

|  | Relevant signalling questions | Response | Elaboration/remarks |
| --- | --- | --- | --- |
| Rahamatillah and Isnaini, 2021 | 4.3 Were important co-interventions balanced across intervention groups? | Y |  |
|  | 4.4 Was the intervention implemented successfully for most participants? | Y |  |
|  | 4.5 Did study participants adhere to the assigned intervention regimen? | PY |  |
| Response to domain 4 | Low risk of bias | - | The important co-interventions were balanced across intervention groups and there were no deviations from the intended interventions (in terms of implementation of adherence) that were likely to impact on the outcome. |

Domain 5: Bias due to missing data

|  | Relevant signalling questions | Response | Elaboration/remarks |
| --- | --- | --- | --- |
| Rahamatillah and Isnaini, 2021 | 5.1 Were outcome data available for all, or nearly all, participants? | Y |  |
|  | 5.2 Were participants excluded due to missing data on intervention status? | Y |  |
|  | 5.3 Were participants excluded due to missing data on other variables needed for the analysis? | PY |  |
|  | 5.4 Are proportion of participants and reasons for missing data similar across interventions? | NI |  |
| Response to domain 5 | Low risk of bias | - | Data were reasonably complete. |

Domain 6: Bias in measurement of outcome

|  | Relevant signalling questions | Response | Elaboration/remarks |
| --- | --- | --- | --- |
| Rahamatillah and Isnaini, 2021 | 6.1 Could the outcome measured have been influenced by knowledge of the intervention received? | Y |  |
|  | 6.2 Were outcome assessors aware of the intervention received by the study participants? | PY |  |
|  | 6.3 Were the methods of outcome assessment comparable across intervention groups? | Y |  |
|  | 6.4 Were any systematic errors in measurement of the outcomes related to intervention received? | NI |  |
| Response to domain 6 | Low risk of bias | - | The methods of outcome assessment were comparable across intervention groups. |

Domain 7: Bias in selection of the reported results

|  | Relevant signalling questions | Response | Elaboration/remarks |
| --- | --- | --- | --- |
| Rahamatillah and Isnaini, 2021 | 7.1 Is the reported effect estimate likely to be selected, on the basis of the results, from multiple outcome measurements within the outcome domain? | Y |  |
|  | 7.2 Is the reported effect estimate likely to be selected, on the basis of the results, from multiple analyses of the intervention outcome relationship? | PY |  |
|  | 7.3 Is the reported effect estimate likely to be selected, on the basis of the results, from different subroups? | Y |  |
| Response to domain 7 | moderate risk of bias | - | The outcome measurement and analyses are consistent with an a *priori* plan. |

Risk of bias assessment: Secondary Outcome 5 (Duration of hospitalisation)

Domain 1: Bias due to confounding

|  | Relevant signalling questions | Response | Elaboration/remarks |
| --- | --- | --- | --- |
| Tan et al., 2020 | 1.1 Is there potential for confounding of the effect of intervention in this study? | Y | Confounding such as those due to comorbidities, medications, and disease severity etc. |
|  | Determine whether there is need to assess time-varying confounding | N |  |
|  | 1.2 Was the analysis based on splitting the participants’ follow-up time according to the intervention received? | N |  |
|  | 1.4 Did the authors use an appropriate analysis method that controlled for all the important confounding domains? | N |  |
|  | 1.5 Were confounding domains that were controlled for measured validly and reliably by the variables available in this study? | NI |  |
|  | Did the authors control for any post-intervention variables that could have been affected by the intervention? | N |  |
| Response for confounding domain | Serious risk of bias | - | At least one known important confounding domain was not appropriately measured or not controlled for. |

Domain 2: Bias in selection of participants into the study

|  | Relevant signalling questions | Response | Elaboration/remarks |
| --- | --- | --- | --- |
| Tan et al., 2020 | 2.1 Was selection of participants into the study (or into the analysis) based on participant characteristics observed after the start of intervention? | PN |  |
|  | 2.4 Do start of follow-up and start of intervention coincide for most participants? | PY |  |
| Response for domain 2 | Low risk of bias | - | All eligible participants were included in the study; and each participants’ start of follow-up and start of intervention coincided. |

Domain 3: Bias in classification of intervention

|  | Relevant signalling questions | Response | Elaboration/remarks |
| --- | --- | --- | --- |
| Tan et al., 2020 | 3.1 Were intervention groups clearly defined? | Y |  |
|  | 3.2 Was the information used to define the intervention groups recorded at the start of the intervention? | Y |  |
|  | 3.3 Could classification of intervention status have been affected by knowledge of the outcome or risk of the outcome? | PY |  |
| Response to domain 3 | Low risk of bias | - | Intervention status is well defined and intervention definition is based solely on information collected at the time of intervention. |

Domain 4: Bias due to deviation from intended intervention

|  | Relevant signalling questions | Response | Elaboration/remarks |
| --- | --- | --- | --- |
| Tan et al., 2020 | 4.3 Were important co-interventions balanced across intervention groups? | PY |  |
|  | 4.4 Was the intervention implemented successfully for most participants? | PY |  |
|  | 4.5 Did study participants adhere to the assigned intervention regimen? | PY |  |
| Response to domain 4 | Low risk of bias | - | The important co-interventions were balanced across intervention groups and there were no deviations from the intended interventions (in terms of implementation of adherence) that were likely to impact on the outcome. |

Domain 5: Bias due to missing data

|  | Relevant signalling questions | Response | Elaboration/remarks |
| --- | --- | --- | --- |
| Tan et al., 2020 | 5.1 Were outcome data available for all, or nearly all, participants? | Y |  |
|  | 5.2 Were participants excluded due to missing data on intervention status? | PY |  |
|  | 5.3 Were participants excluded due to missing data on other variables needed for the analysis? | PY |  |
|  | 5.4 Are proportion of participants and reasons for missing data similar across interventions? | NI |  |
| Response to domain 5 | Low risk of bias | - | Data were reasonably complete. |

Domain 6: Bias in measurement of outcome

|  | Relevant signalling questions | Response | Elaboration/remarks |
| --- | --- | --- | --- |
| Tan et al., 2020 | 6.1 Could the outcome measured have been influenced by knowledge of the intervention received? | Y |  |
|  | 6.2 Were outcome assessors aware of the intervention received by the study participants? | PY |  |
|  | 6.3 Were the methods of outcome assessment comparable across intervention groups? | Y |  |
|  | 6.4 Were any systematic errors in measurement of the outcomes related to intervention received? | NI |  |
| Response to domain 6 | Low risk of bias | - | The methods of outcome assessment were comparable across intervention groups. |

Domain 7: Bias in selection of the reported results

|  | Relevant signalling questions | Response | Elaboration/remarks |
| --- | --- | --- | --- |
| Tan et al., 2020 | 7.1 Is the reported effect estimate likely to be selected, on the basis of the results, from multiple outcome measurements within the outcome domain? | Y |  |
|  | 7.2 Is the reported effect estimate likely to be selected, on the basis of the results, from multiple analyses of the intervention outcome relationship? | PY |  |
|  | 7.3 Is the reported effect estimate likely to be selected, on the basis of the results, from different subroups? | Y |  |
| Response to domain 7 | moderate risk of bias | - | The outcome measurement and analyses are consistent with an a *priori* plan. |

Domain 1: Bias due to confounding

|  | Relevant signalling questions | Response | Elaboration/remarks |
| --- | --- | --- | --- |
| Vahedi et al., 2020 | 1.1 Is there potential for confounding of the effect of intervention in this study? | Y | Confounding such as those due to comorbidities, medications, and disease severity etc. |
|  | Determine whether there is need to assess time-varying confounding | N |  |
|  | 1.2 Was the analysis based on splitting the participants’ follow-up time according to the intervention received? | N |  |
|  | 1.4 Did the authors use an appropriate analysis method that controlled for all the important confounding domains? | N |  |
|  | 1.5 Were confounding domains that were controlled for measured validly and reliably by the variables available in this study? | NI |  |
|  | Did the authors control for any post-intervention variables that could have been affected by the intervention? | N |  |
| Response for confounding domain | Serious risk of bias | - | At least one known important confounding domain was not appropriately measured or not controlled for. |

Domain 2: Bias in selection of participants into the study

|  | Relevant signalling questions | Response | Elaboration/remarks |
| --- | --- | --- | --- |
| Vahedi et al., 2020 | 2.1 Was selection of participants into the study (or into the analysis) based on participant characteristics observed after the start of intervention? | PN |  |
|  | 2.4 Do start of follow-up and start of intervention coincide for most participants? | PY |  |
| Response for domain 2 | Low risk of bias | - | All eligible participants were included in the study; and each participants’ start of follow-up and start of intervention coincided. |

Domain 3: Bias in classification of intervention

|  | Relevant signalling questions | Response | Elaboration/remarks |
| --- | --- | --- | --- |
| Vahedi et al., 2020 | 3.1 Were intervention groups clearly defined? | Y |  |
|  | 3.2 Was the information used to define the intervention groups recorded at the start of the intervention? | Y |  |
|  | 3.3 Could classification of intervention status have been affected by knowledge of the outcome or risk of the outcome? | PY |  |
| Response to domain 3 | Low risk of bias | - | Intervention status is well defined and intervention definition is based solely on information collected at the time of intervention. |

Domain 4: Bias due to deviation from intended intervention

|  | Relevant signalling questions | Response | Elaboration/remarks |
| --- | --- | --- | --- |
| Vahedi et al., 2020 | 4.3 Were important co-interventions balanced across intervention groups? | PY |  |
|  | 4.4 Was the intervention implemented successfully for most participants? | PY |  |
|  | 4.5 Did study participants adhere to the assigned intervention regimen? | PY |  |
| Response to domain 4 | Low risk of bias | - | The important co-interventions were balanced across intervention groups and there were no deviations from the intended interventions (in terms of implementation of adherence) that were likely to impact on the outcome. |

Domain 5: Bias due to missing data

|  | Relevant signalling questions | Response | Elaboration/remarks |
| --- | --- | --- | --- |
| Vahedi et al., 2020 | 5.1 Were outcome data available for all, or nearly all, participants? | Y |  |
|  | 5.2 Were participants excluded due to missing data on intervention status? | PY |  |
|  | 5.3 Were participants excluded due to missing data on other variables needed for the analysis? | PY |  |
|  | 5.4 Are proportion of participants and reasons for missing data similar across interventions? | NI |  |
| Response to domain 5 | Low risk of bias | - | Data were reasonably complete. |

Domain 6: Bias in measurement of outcome

|  | Relevant signalling questions | Response | Elaboration/remarks |
| --- | --- | --- | --- |
| Vahedi et al., 2020 | 6.1 Could the outcome measured have been influenced by knowledge of the intervention received? | Y |  |
|  | 6.2 Were outcome assessors aware of the intervention received by the study participants? | PY |  |
|  | 6.3 Were the methods of outcome assessment comparable across intervention groups? | Y |  |
|  | 6.4 Were any systematic errors in measurement of the outcomes related to intervention received? | NI |  |
| Response to domain 6 | Low risk of bias | - | The methods of outcome assessment were comparable across intervention groups. |

Domain 7: Bias in selection of the reported results

|  | Relevant signalling questions | Response | Elaboration/remarks |
| --- | --- | --- | --- |
| Vahedi et al., 2020 | 7.1 Is the reported effect estimate likely to be selected, on the basis of the results, from multiple outcome measurements within the outcome domain? | Y |  |
|  | 7.2 Is the reported effect estimate likely to be selected, on the basis of the results, from multiple analyses of the intervention outcome relationship? | PY |  |
|  | 7.3 Is the reported effect estimate likely to be selected, on the basis of the results, from different subroups? | Y |  |
| Response to domain 7 | moderate risk of bias | - | The outcome measurement and analyses are consistent with an a *priori* plan. |

Domain 1: Bias due to confounding

|  | Relevant signalling questions | Response | Elaboration/remarks |
| --- | --- | --- | --- |
| Haghjoo et al., 2021 | 1.1 Is there potential for confounding of the effect of intervention in this study? | Y | Confounding such as those due to comorbidities, medications, and disease severity etc. |
|  | Determine whether there is need to assess time-varying confounding | N |  |
|  | 1.2 Was the analysis based on splitting the participants’ follow-up time according to the intervention received? | N |  |
|  | 1.4 Did the authors use an appropriate analysis method that controlled for all the important confounding domains? | N | Authors used logistic regression analysis. |
|  | 1.5 Were confounding domains that were controlled for measured validly and reliably by the variables available in this study? | NI |  |
|  | Did the authors control for any post-intervention variables that could have been affected by the intervention? | N |  |
| Response for confounding domain | Moderate risk of bias | - | Confounding is expected but all known important confounding domains were appropriately measured and controlled for; and reliability and validity of important domains were sufficient, such that we do not expect serious residual confounding. |

Domain 2: Bias in selection of participants into the study

|  | Relevant signalling questions | Response | Elaboration/remarks |
| --- | --- | --- | --- |
| Haghjoo et al., 2021 | 2.1 Was selection of participants into the study (or into the analysis) based on participant characteristics observed after the start of intervention? | PN |  |
|  | 2.4 Do start of follow-up and start of intervention coincide for most participants? | PY |  |
| Response for domain 2 | Low risk of bias | - | All eligible participants were included in the study; and each participants’ start of follow-up and start of intervention coincided. |

Domain 3: Bias in classification of intervention

|  | Relevant signalling questions | Response | Elaboration/remarks |
| --- | --- | --- | --- |
| Haghjoo et al., 2021 | 3.1 Were intervention groups clearly defined? | Y |  |
|  | 3.2 Was the information used to define the intervention groups recorded at the start of the intervention? | Y |  |
|  | 3.3 Could classification of intervention status have been affected by knowledge of the outcome or risk of the outcome? | PY |  |
| Response to domain 3 | Low risk of bias | - | Intervention status is well defined and intervention definition is based solely on information collected at the time of intervention. |

Domain 4: Bias due to deviation from intended intervention

|  | Relevant signalling questions | Response | Elaboration/remarks |
| --- | --- | --- | --- |
| Haghjoo et al., 2021 | 4.3 Were important co-interventions balanced across intervention groups? | PY |  |
|  | 4.4 Was the intervention implemented successfully for most participants? | PY |  |
|  | 4.5 Did study participants adhere to the assigned intervention regimen? | PY |  |
| Response to domain 4 | Low risk of bias | - | The important co-interventions were balanced across intervention groups and there were no deviations from the intended interventions (in terms of implementation of adherence) that were likely to impact on the outcome. |

Domain 5: Bias due to missing data

|  | Relevant signalling questions | Response | Elaboration/remarks |
| --- | --- | --- | --- |
| Haghjoo et al., 2021 | 5.1 Were outcome data available for all, or nearly all, participants? | Y |  |
|  | 5.2 Were participants excluded due to missing data on intervention status? | PY |  |
|  | 5.3 Were participants excluded due to missing data on other variables needed for the analysis? | PY |  |
|  | 5.4 Are proportion of participants and reasons for missing data similar across interventions? | NI |  |
| Response to domain 5 | Low risk of bias | - | Data were reasonably complete. |

Domain 6: Bias in measurement of outcome

|  | Relevant signalling questions | Response | Elaboration/remarks |
| --- | --- | --- | --- |
| Haghjoo et al., 2021 | 6.1 Could the outcome measured have been influenced by knowledge of the intervention received? | Y |  |
|  | 6.2 Were outcome assessors aware of the intervention received by the study participants? | PY |  |
|  | 6.3 Were the methods of outcome assessment comparable across intervention groups? | Y |  |
|  | 6.4 Were any systematic errors in measurement of the outcomes related to intervention received? | NI |  |
| Response to domain 6 | Low risk of bias | - | The methods of outcome assessment were comparable across intervention groups. |

Domain 7: Bias in selection of the reported results

|  | Relevant signalling questions | Response | Elaboration/remarks |
| --- | --- | --- | --- |
| Haghjoo et al., 2021 | 7.1 Is the reported effect estimate likely to be selected, on the basis of the results, from multiple outcome measurements within the outcome domain? | Y |  |
|  | 7.2 Is the reported effect estimate likely to be selected, on the basis of the results, from multiple analyses of the intervention outcome relationship? | PY |  |
|  | 7.3 Is the reported effect estimate likely to be selected, on the basis of the results, from different subroups? | Y |  |
| Response to domain 7 | moderate risk of bias | - | The outcome measurement and analyses are consistent with an a *priori* plan. |

Interpretation of risk of bias judgments [[1](#_ENREF_1)]

| Response options | Interpretation |
| --- | --- |
| Low risk of bias | The study is comparable to a well-performed randomised trial with regards to this bias domain. |
| Moderate risk of bias | The study is sound for a non-randomised study with regards to this bias domain but can not be considered comparable to a well-performed randomised trial. |
| Serious risk of bias | The study has some important problems with regards to this bias domain. |
| Critical risk of bias | The study is too problematic in this domain of bias to prove any useful evidence. |
| No information | No information on which to base a judgement risk of bias for this domain. |

**References**

1. Sterne, J.A., et al., *ROBINS-I: a tool for assessing risk of bias in non-randomised studies of interventions.* bmj, 2016. **355**.
