# Supplementary material for: Systematic review and meta-analysis of the efficacy and safety of oseltamivir (Tamiflu) in the treatment of Coronavirus Disease 2019 (COVID-19)
Source: PLoS One. 2022 Dec 1;17(12):e0277206. doi: 10.1371/journal.pone.0277206 (PMC9714710; doi:10.1371/journal.pone.0277206)
Supplement: S7 File — (DOCX) [file pone.0277206.s007.docx]

S7 File

Sensitivity analysis using Fixed effect model for the primary outcome


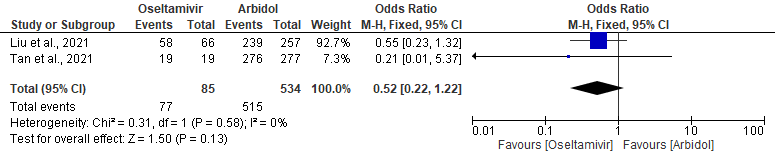


Figure 1: graphical presentation of the meta-analysis comparing the survival of the Oseltamivir group to the Arbidol group


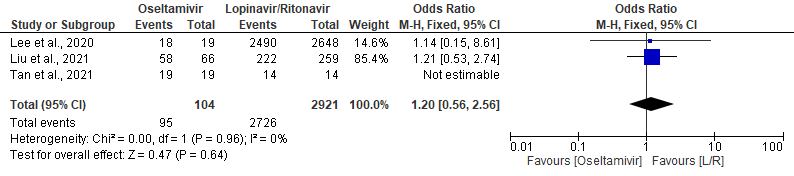


Figure 2: Forest plot of the meta-analysis comparing the survival of the Oseltamivir group to the Lopinavir/Ritonavir group

Figure 3: Prediction interval plot comparing Oseltamivir to other drugs.

Figure 4: PI plot for the sensitivity analysis.

Figure 5: PI plot for duration of hospitalisation.

Figure 6: PI plot for duration of hospitalisation sensitivity
