# Supplementary material for: Systematic review and meta-analysis of the efficacy and safety of oseltamivir (Tamiflu) in the treatment of Coronavirus Disease 2019 (COVID-19)
Source: PLoS One. 2022 Dec 1;17(12):e0277206. doi: 10.1371/journal.pone.0277206 (PMC9714710; doi:10.1371/journal.pone.0277206)
Supplement: S8 File — (DOCX) [file pone.0277206.s008.docx]

**S8 File**

**Summary of TSA (Odds Ratio)**


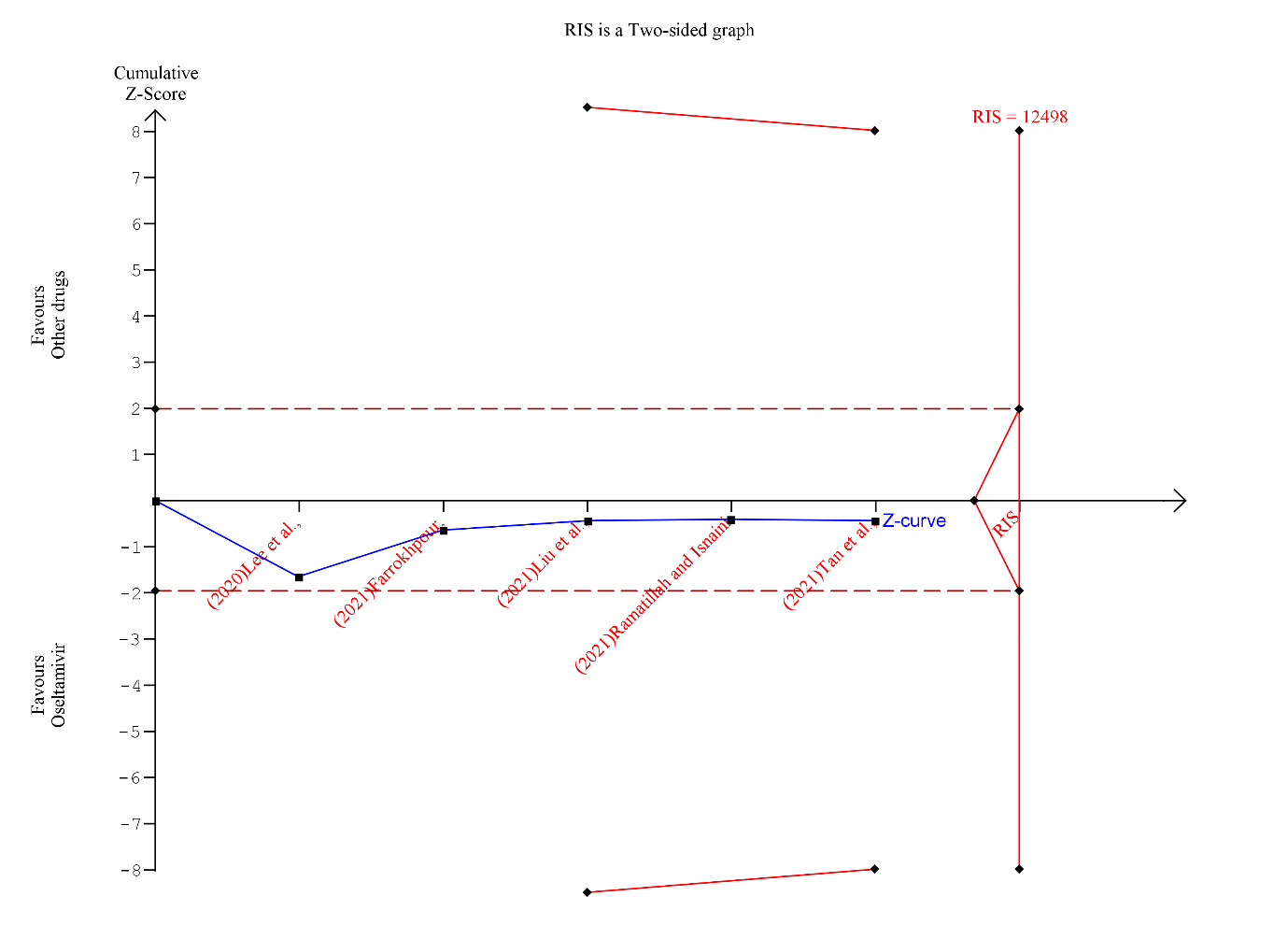


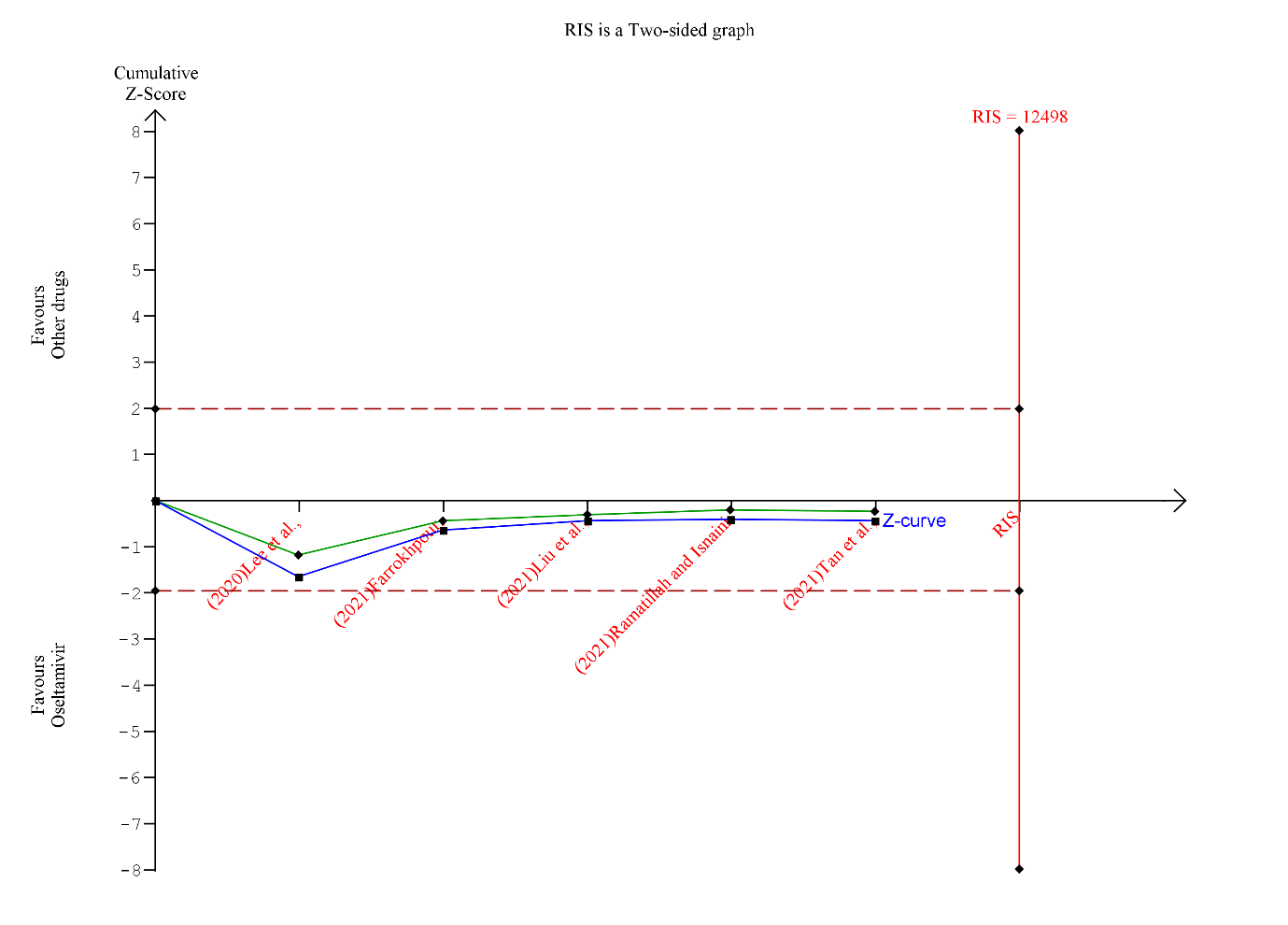


**Meta-analysis**

Identifier : Survival of COVID-19 patients
Group 1 Label : Oseltamivir
Group 2 Label : Other drugs
Outcome Type : Postive
Comment :
Effect Measure : Odds Ratio
Effect Model : Random-effects (SJ)
Zero Event handling : Empirical (shared value: 0.01)
Zero-event Trials are included

Pooled Effect : 1.7 (C.I: 0.18 to 15.88)
based on conventional 95%
P-value : 0.64
Heterogeneity (Q) : 15.06
Heterogeneity (Q) P-value : 0.0046
Inconsistency (I²) : 0.73
Diversity (D²) : 0.9

## Boundaries

Name: Conventional (Conventional)
Type: Two-sided
Type 1 Error: 5.0

Name: RIS (Alpha-spending)
Type: Two-sided
Type 1 Error: 5.0%
Alpha Spending: O'Brien-Fleming
Information Axis: Sample Size
IS Type: Estimate
Power: 80.0%
Effect Type Intervention: RRR User Defined (1.95%)
Heterogeneity Correction: User Defined (0.45%)
O'Brien Fleming

Name: LIL (LIL)
Type: Two-sided
Type 1 Error: 5.0
Penalty: 2.0

## Trials

Name : (2020)Lee et al.,
Effect measure : 36.0
Standard Error : 1.0865337342004415
Weight : 0.21838594956282933
Variance : 4.579049165030195
Weight percentage : 28.33% (0.2833423789894229)


Name : (2021)Farrokhpour,
Effect measure : 0.49382716049382713
Standard Error : 0.682791112789046
Weight : 0.25875247631685694
Variance : 3.8646973131783438
Weight percentage : 33.57% (0.3357154723359608)


Name : (2021)Liu et al.,
Effect measure : 0.5460251046025104
Standard Error : 0.4494230026203715
Weight : 0.2777411587818443
Variance : 3.60047464475895
Weight percentage : 36.04% (0.36035212352288315)


Name : (2021)Ramatillah and Isnaini
Effect measure : 0.003929775775420716
Standard Error : 11.75371600257984
Weight : 0.007064724645097582
Variance : 141.54833347877602
Weight percentage : 0.92% (0.009166047045857493)


Name : (2021)Tan et al.,
Effect measure : 7.521601877897703
Standard Error : 10.496334475415537
Weight : 0.008805023503137109
Variance : 113.57153102927138
Weight percentage : 1.14% (0.011423978105875763)
