# Supplementary material for: Systematic review and meta-analysis of the efficacy and safety of oseltamivir (Tamiflu) in the treatment of Coronavirus Disease 2019 (COVID-19)
Source: PLoS One. 2022 Dec 1;17(12):e0277206. doi: 10.1371/journal.pone.0277206 (PMC9714710; doi:10.1371/journal.pone.0277206)
Supplement: S9 File — (DOCX) [file pone.0277206.s009.docx]

S9 File

**Summary for the duration of hospitalisation of COVID-19 patients**


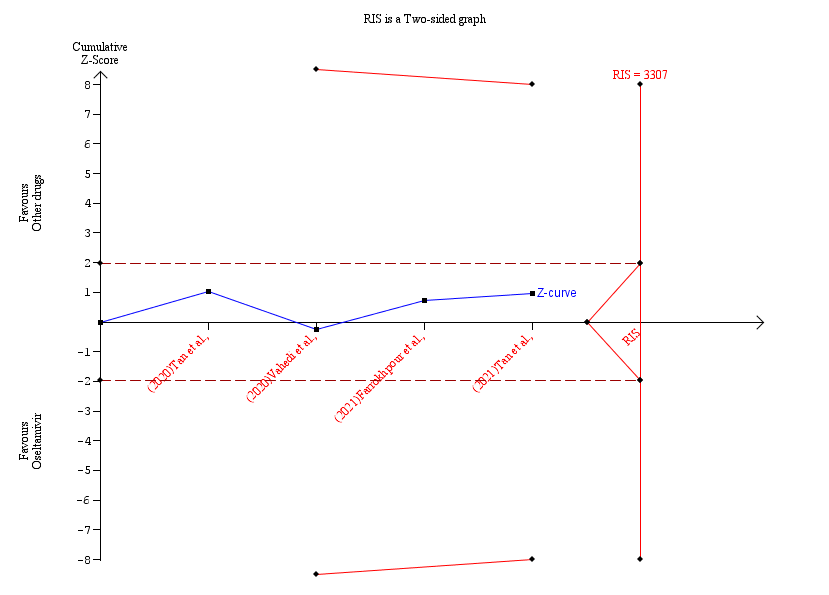


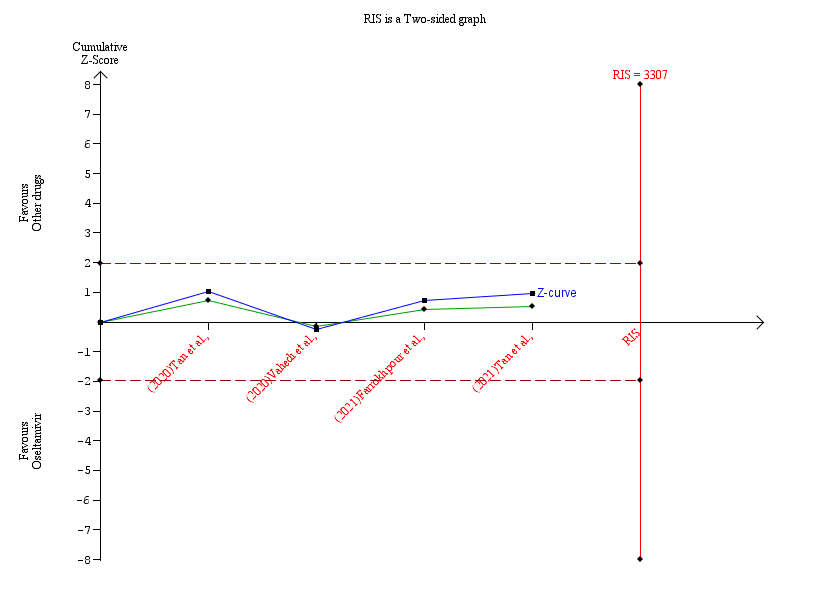


**Meta-analysis**

Identifier : Duration of hospitalisation
Group 1 Label : Oseltamivir
Group 2 Label : Other drugs
Outcome Type : Postive
Comment :
Effect Measure : Mean Difference
Effect Model : Random-effects (SJ)
Zero Event handling : null (shared value: 0.0)
Zero-event Trials are not included

Pooled Effect : -2.78 (C.I: -8.46 to 2.89)
based on conventional 95%
P-value : 0.3366
Heterogeneity (Q) : 18.78
Heterogeneity (Q) P-value : 3.0E-4
Inconsistency (I²) : 0.84
Diversity (D²) : 0.93

## Boundaries

Name: Conventional (Conventional)
Type: Two-sided
Type 1 Error: 5.0

Name: RIS (Sequential Continuous)
Type: Two-sided
Type 1 Error: 5.0
Alpha Spending: O'Brien-Fleming
Information Axis: Sample Size
IS Type: Estimate
Power: 80.0
Effect Type Mean: Low Bias Based
Effect Type Variance: Low Bias Based
Heterogeneity Correction: Variance Based
O'Brien Fleming

Name: LIL (LIL)
Type: Two-sided
Type 1 Error: 5.0
Penalty: 2.0

## Trials

Name : (2020)Tan et al.,
Effect Measure : -3.0
Weight : 0.03307456545853933
Variance : 8.42532747747748
Weight Percentage : 27.72% (0.2771865867774395)


Name : (2020)Vahedi et al.,
Effect Measure : 2.96
Weight : 0.04452666567668214
Variance : 0.6490666666666667
Weight Percentage : 37.32% (0.3731627100277785)


Name : (2021)Farrokhpour et al.,
Effect Measure : -8.7
Weight : 0.032810263243302024
Variance : 8.668882416666666
Weight Percentage : 27.5% (0.27497156058121686)


Name : (2021)Tan et al.,
Effect Measure : -8.87
Weight : 0.008910893631166816
Variance : 90.41280451127818
Weight Percentage : 7.47% (0.07467914261356513)
