# Supplementary material for: Systematic review and meta-analysis of the efficacy and safety of oseltamivir (Tamiflu) in the treatment of Coronavirus Disease 2019 (COVID-19)
Source: PLoS One. 2022 Dec 1;17(12):e0277206. doi: 10.1371/journal.pone.0277206 (PMC9714710; doi:10.1371/journal.pone.0277206)
Supplement: S10 File — (DOCX) [file pone.0277206.s010.docx]

**S10 File**

Table 1: Grading of Recommendations, Assessment, Development and Evaluations (GRADE) [[1](#_ENREF_1), [2](#_ENREF_2)]

| Outcome | Studies assessed | Domain | Explanation | Decision |
| --- | --- | --- | --- | --- |
| Patient recovery | Lee et al., 2020 | Risk of bias | One study (Tan et al., 2021) had serious RoB response in D1 of ROBINS-I tool assessment. | Serious risk of bias |
|  | Liu et al., 2021 | Imprecision | Adequate sample size  Adequate number of events.  Wide 95% confidence interval (CI) that precludes OR of 1.0. | Imprecision not a concern |
|  | Tan et al., 2021 | Inconsistency | Relative low variation of point estimates from study to study.  Presence of overlapping CI.  I^2^ is 74% with P value of 0.004. However, in sensitivity analysis after the removal of one study the I^2^ reduced to 0% with a P value of 0.81. | Inconsistency not serious. |
|  | Farrokhpour et al., 2021 | Indirectness | Population: appropriate for all studies.  Intervention: appropriate intervention used in all included studies.  Comparison: interventions are appropriately compared with alternatives.  Outcome: assessment of the appropriate outcome in all included studies. | No concern for indirectness |
|  | Rahamatillah and Isnaini, 2021 | Publication bias | Although there are only 5 included studies (while the rule of thumb requires ≥10 studies for a funnel plot), the funnel plot for the 5 studies shows a nearly symmetrical distribution of the individual studies around the point estimate (OR: 0.88). Indicating that publication bias is not likely. | Publication bias undetected |
|  | | | | |
| Virological response | Tan et al., 2021 | Risk of bias | Serious RoB response in D1 of ROBINS-I tool. | Serious risk of bias |
|  |  | Imprecision | The total sample size in the study is 333 which is less than the recommended 400. | Serious imprecision |
|  |  | Inconsistency | Only one study | No inconsistency |
|  |  | Indirectness | The study outcome is appropriate with the objective of the review and intervention was appropriately compared and in the right population | Indirectness not a concern |
|  |  | Publication bias | This systematic review did a thorough and comprehensive literature search. Thus, publication bias not likely. | Undetected |
|  | | | | |
| Laboratory response | Vahedi et., 2020 | Risk of bias | Serious RoB response in D1 of ROBINS-I tool. | Serious risk of bias |
|  |  | Imprecision | 60 participants were enrolled in this study | Serious imprecision |
|  |  | Inconsistency | One study | No inconsistency |
|  |  | Indirectness | The study outcome is appropriate with the objective of the review and intervention was appropriately compared and in the right population | Indirectness not a concern |
|  |  | Publication bias | This systematic review did a thorough and comprehensive literature search. Thus, publication bias not likely. | Undetected |
|  |  |  |  |  |
| Radiologic response | Vahedi et., 2020  Tan et al., 2020 | Risk of bias | Serious RoB response in D1 of ROBINS-I tool in both studies. | Serious risk of bias |
|  |  | Imprecision | 60 participants were enrolled in the Vahedi study while 326 were enrolled in the tan study. | Serious imprecision |
|  |  | Inconsistency | The results of the two studies were inconsistent. | Inconsistency |
|  |  | Indirectness | The study outcome is appropriate with the objective of the review and intervention was appropriately compared and in the right population | Indirectness not a concern |
|  |  | Publication bias | This systematic review did a thorough and comprehensive literature search. Thus, publication bias not likely. | Undetected |
|  | | | | |
| Duration of hospitalisation | Tan et al., 2020 | Risk of bias | Three studies (Tan et al., 2020, Vahedi et al., 2020, & Tan et al., 2021) had serious RoB response in D1 of ROBINS-I tool assessment. | Serious risk off bias |
|  | Vahedi et al., 2020 | Imprecision | Two out of the four studies have the lower boundaries of their CI below the threshold. While one of the studies has the entire CI and point estimate below the threshold. | Serious imprecision |
|  | Tan et al., 2021 | Inconsistency | There is significant overlap of Cis across three of the four studies. The I2 is 84% (p=0.0003). However, when one of the studies was removed (sensitivity analysis), the I2 reduced to 0%. | No concern about inconsistency. |
|  | Farrokhpour et al., 2021 | Indirectness | Appropriate PICO for the outcome assessed | No concern for indirectness |
|  |  | Publication bias | The funnel plot for the 4 studies shows a nearly symmetrical distribution of the individual studies around the point estimate (MD: ). Indicating that publication bias is not likely. | Undetected |
|  |  |  |  |  |
| Safety evaluation | Haghjoo et al., 2021 | Risk of bias | Confounding measured | Moderate risk of bias |
|  |  | Imprecision | Number of participants in the study (sample size) is 2403 which is higher than the recommended 400. | Imprecision not a concern |
|  |  | Inconsistency | Only one study | No concern about inconsistency |
|  |  | Indirectness | The study outcome is appropriate with the objective of the review and intervention was appropriately compared and in the right population | Indirectness not a concern |
|  |  | Publication bias | This systematic review did a thorough and comprehensive literature search. Thus, publication bias not likely. | Undetected |

Table 2: Interpretation of GRADE Quality of Evidence [[1](#_ENREF_1)]

| S/N | Level | Explanation |
| --- | --- | --- |
| 1 | High | We are very confident that the true effect lies close to that of the estimate of effect. |
| 2 | Moderate | We are moderately confident in the effect estimate; the true effect is likely to be close to the estimate of the effect, but there is possibility that it is substantially different. |
| 3 | Low | Our confidence in the effect estimate is limited; the true effect may be substantially different from the estimate of the effect. |
| 4 | Very low | We have very little confidence in the effect estimate; the true effect is likely to be substantially different from the estimate effect. |


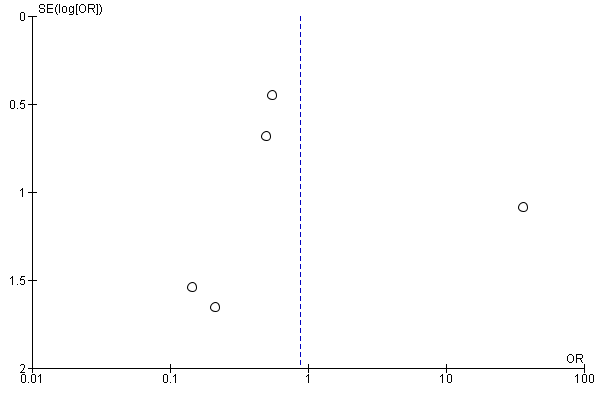


Figure 1: Funnel plot for the primary outcome – Patient recovery.


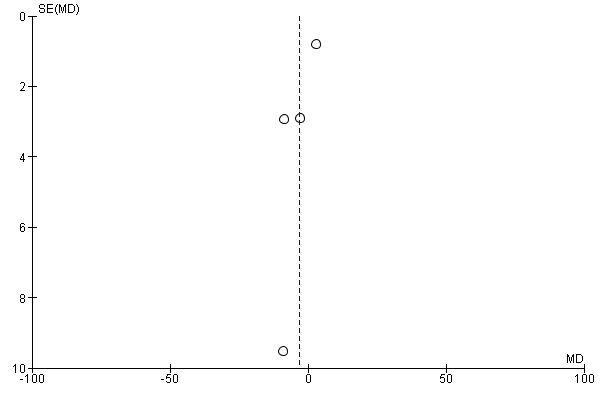


Figure 2: Funnel plot for the duration of hospitalisation outcome

**References**

1. Schünemann, H., et al., *GRADE handbook for grading quality of evidence and strength of recommendations. Updated October 2013. The GRADE Working Group, 2013.* Available from guidelinedevelopment. org/handbook, 2019.

2. Murad, M.H., et al., *Rating the certainty in evidence in the absence of a single estimate of effect.* BMJ Evidence-Based Medicine, 2017. **22**(3): p. 85-87.
