# Supplementary material for: Systematic review and meta-analysis of the efficacy and safety of oseltamivir (Tamiflu) in the treatment of Coronavirus Disease 2019 (COVID-19)
Source: PLoS One. 2022 Dec 1;17(12):e0277206. doi: 10.1371/journal.pone.0277206 (PMC9714710; doi:10.1371/journal.pone.0277206)
Supplement: S2 Table — (DOCX) [file pone.0277206.s012.docx]

**S1 Table**

Articles screened for full text

| S/N | Study | Decision | Reason |
| --- | --- | --- | --- |
| 1 | Akram, J., Azhar, S., Shahzad, M., Latif, W., & Khan, K. S. (2020). Pakistan Randomized and Observational Trial to Evaluate Coronavirus Treatment (PROTECT) of Hydroxychloroquine, Oseltamivir and Azithromycin to treat newly diagnosed patients with COVID-19 infection who have no comorbidities like diabetes mellitus: A structured summary of a study protocol for a randomized controlled trial. *Trials, 21*(1), 1-3. | Exclude | Letter of trial protocol |
| 2 | Bérard, A., Sheehy, O., Zhao, J.-P., Vinet, E., Quach, C., Kassai, B., & Bernatsky, S. (2021). Available medications used as potential therapeutics for COVID-19: What are the known safety profiles in pregnancy. *PloS one, 16*(5), e0251746. | Exclude | Wrong outcome |
| 3 | Cheng, F., Li, Q., Han, Y., Shi, C., Wu, S., Xu, Q., . . . Zhang, Y. (2020). Analysis of influencing factors and pharmaceutical care of patients with COVID-19 in Fangcang Shelter Hospital. *Infection and Drug Resistance, 13*, 3443. | Exclude | Patients’ outcomes merged not separated |
| 4 | Chiba, S. (2021). Effect of early oseltamivir on outpatients without hypoxia with suspected COVID-19. *Wiener klinische Wochenschrift, 133*(7), 292-297. | Exclude | Suspected COVID-19 study |
| 5 | Coenen, S., van Der Velden, A. W., Cianci, D., Goossens, H., Bongard, E., Saville, B. R., . . . Verheij, T. J. (2020). Oseltamivir for coronavirus illness: post-hoc exploratory analysis of an open-label, pragmatic, randomised controlled trial in European primary care from 2016 to 2018. *British Journal of General Practice, 70*(696), e444-e449. | Exclude | Non-SARS-CoV-2 Coronaviruses study |
| 6 | Costanzo, M., De Giglio, M. A., & Roviello, G. N. (2020). SARS-CoV-2: recent reports on antiviral therapies based on lopinavir/ritonavir, darunavir/umifenovir, hydroxychloroquine, remdesivir, favipiravir and other drugs for the treatment of the new coronavirus. *Current medicinal chemistry, 27*(27), 4536-4541. | Exclude | Perspective (review) article |
| 7 | Dabbous, H. M., El-Sayed, M. H., El Assal, G., Elghazaly, H., Ebeid, F. F., Sherief, A. F., . . . Riad, A. R. (2021). Safety and efficacy of favipiravir versus hydroxychloroquine in management of COVID-19: A randomised controlled trial. *Scientific Reports, 11*(1), 1-7. | Exclude | Retracted article |
| 8 | Ding, Q., Lu, P., Fan, Y., Xia, Y., & Liu, M. (2020). The clinical characteristics of pneumonia patients coinfected with 2019 novel coronavirus and influenza virus in Wuhan, China. *Journal of medical virology, 92*(9), 1549-1555. | Exclude | Co-infection of Influenza and COVID-19 |
| 9 | Farrokhpour, M., Rezaie, N., Moradi, N., Rad, F. G., Izadi, S., Azimi, M., . . . Makiani, M. J. (2021). Infliximab and intravenous Gammaglobulin in hospitalized severe COVID-19 patients in intensive care unit. *Archives of Iranian medicine, 24*(2), 139-143. | Include |  |
| 10 | Fricke-Galindo, I., & Falfán-Valencia, R. (2021). Pharmacogenetics Approach for the Improvement of COVID-19 Treatment. *Viruses, 13*(3), 413. | Exclude | Review article |
| 11 | Haghjoo, M., Golipra, R., Kheirkhah, J., Golabchi, A., Shahabi, J., Oni‐Heris, S., . . . Khatami, M. (2021). Effect of COVID‐19 medications on corrected QT interval and induction of torsade de pointes: Results of a multicenter national survey. *International journal of clinical practice*, e14182. | Include |  |
| 12 | Hu, F., Yin, G., Chen, Y., Song, J., Ye, M., Liu, J., . . . Zhang, Y. (2020). Corticosteroid, oseltamivir and delayed admission are independent risk factors for prolonged viral shedding in patients with Coronavirus Disease 2019. *The clinical respiratory journal, 14*(11), 1067-1075. | Exclude | Wrong outcome presentation |
| 13 | Ilgın, B. U., Koyuncu, İ. M. A., & Kızıltunç, E. (2021). Effect of triple antimicrobial therapy on electrocardiography parameters in patients with mild-to-moderate coronavirus disease 2019. *Anatolian Journal of Cardiology, 25*(3), 184. | Exclude | Both groups on Oseltamivir |
| 14 | Lee, S.-G., Park, G. U., Moon, Y. R., & Sung, K. (2020). Clinical characteristics and risk factors for fatality and severity in patients with coronavirus disease in Korea: A nationwide population-based retrospective study using the Korean Health Insurance Review and Assessment Service (HIRA) database. *International Journal of Environmental Research and Public Health, 17*(22), 8559. | Include |  |
| 15 | Liu, Q., Fang, X., Tian, L., Vankadari, N., Chen, X., Wang, K., . . . Shen, L. (2021). Arbidol treatment with reduced mortality of adult patients with COVID-19 in Wuhan, China: a retrospective cohort study. *medRxiv*, 2020.2004. 2011.20056523. | Include |  |
| 16 | Louchet, M., Sibiude, J., Peytavin, G., Picone, O., Tréluyer, J.-M., & Mandelbrot, L. (2020). Placental transfer and safety in pregnancy of medications under investigation to treat coronavirus disease 2019. *American journal of obstetrics & gynecology MFM, 2*(3), 100159. | Exclude | Systematic review |
| 17 | Panda, P. K., Bandyopadhyay, A., Singh, B. C., Moirangthem, B., Chikara, G., Saha, S., & Bahurupi, Y. A. (2020). Safety and efficacy of antiviral combination therapy in symptomatic patients of Covid-19 infection-a randomised controlled trial (SEV-COVID Trial): A structured summary of a study protocol for a randomized controlled trial. *Trials, 21*(1), 1-3. | Exclude | Protocol of trial study |
| 18 | Ramatillah, D. L., & Isnaini, S. (2021). Treatment profiles and clinical outcomes of COVID-19 patients at private hospital in Jakarta. *PloS one, 16*(4), e0250147. | Include |  |
| 19 | Tan, J., Yuan, Y., Xu, C., Song, C., Liu, D., Ma, D., & Gao, Q. (2021). A retrospective comparison of drugs against COVID-19. *Virus research, 294*, 198262. doi: https://doi.org/10.1016/j.virusres.2020.198262 | Include |  |
| 20 | Tan, Q., Duan, L., Ma, Y., Wu, F., Huang, Q., Mao, K., . . . Zhou, E. (2020). Is oseltamivir suitable for fighting against COVID-19: In silico assessment, in vitro and retrospective study. *Bioorganic chemistry, 104*, 104257. | Include |  |
| 21 | Vahedi, E., Ghanei, M., Ghazvini, A., Azadi, H., Izadi, M., Panahi, Y., . . . Ghazale, A. H. (2020). The clinical value of two combination regimens in the Management of Patients Suffering from Covid-19 pneumonia: a single centered, retrospective, observational study. *DARU Journal of Pharmaceutical Sciences, 28*(2), 507-516. | Include |  |
| 22 | Yousefi, B., Valizadeh, S., Ghaffari, H., Vahedi, A., Karbalaei, M., & Eslami, M. (2020). A global treatments for coronaviruses including COVID‐19. *Journal of cellular physiology, 235*(12), 9133-9142. | Exclude | Review article |
| 23 | Yu, T., Tian, C., Chu, S., Zhou, H., Zhang, Z., Luo, S., . . . Fan, H. (2020). COVID‐19 patients benefit from early antiviral treatment: A comparative, retrospective study. *Journal of medical virology, 92*(11), 2675-2683. | Exclude | Wrong outcome presentation |
