# Supplementary material for: Systematic review and meta-analysis of the efficacy and safety of oseltamivir (Tamiflu) in the treatment of Coronavirus Disease 2019 (COVID-19)
Source: PLoS One. 2022 Dec 1;17(12):e0277206. doi: 10.1371/journal.pone.0277206 (PMC9714710; doi:10.1371/journal.pone.0277206)
Supplement: S3 Table — (DOCX) [file pone.0277206.s013.docx]

S2 Table

Characteristics of clinical trials studying the efficacy and safety of Oseltamivir monotherapy or combination formulation in patients with COVID-19.

| **ID** | **Title** | **Country** | **Recruiting status** | **Study design** | **Population** | | | | **Characteristics** | | | | **Date** | |
| --- | --- | --- | --- | --- | --- | --- | --- | --- | --- | --- | --- | --- | --- | --- |
|  |  |  |  |  | **Disease condition** | **Enrolment sample size** | **Age** | **Sex** | **Intervention** | **Study type** | **Phase** | **Outcomes** | **Start** | **Proposed completion** |
| NCT04558463 | The Effectivity and Safety  of Favipiravir Compared to  Oseltamivir as Adjuvant Therapy  for COVID-19 | Indonesia | Recruiting | RCT | COVID-19 | 100 | 18-75 years | M/F | Favipiravir  Vs Oseltamivir  75mg | Interventional | 3 | •Clinical radiologic changes  •Percentage of RT-PCR  test conversion  •Adverse event  •Hospital length of stay  (LOS)  •Case Fatality Rate (CFR) | 04/16/2020 | 10/30/2020 |
| NCT04516915 | IMU-838 and Oseltamivir in the  Treatment of COVID-19 | United Kingdom | Recruiting | RCT | COVID-19 | 120 | 18-90 years | M/F | IMU-838  Vs Oseltamivir | Interventional | 2 | •To evaluate whether  time-to-improvement  is significantly better in  IMU-838 plus Oseltamivir  (IONIC Intervention) vs.  Oseltamivir alone in adult  subjects with COVID-19  •To evaluate safety  (number of adverse  events) and tolerability  (laboratory abnormalities)  of IMU 838 + Oseltamivir  vs. Oseltamivir alone  in adult subjects with  COVID-19.  •To determine the effects  of IONIC Intervention on  improvement of at least  two points in clinical status  scale (from 0 to 8; with  8 being no evidence of  clinical infection and 8  being death)  •To assess the effects of  IONIC Intervention vs.  Oseltamivir on the need for  invasive ventilation, renal  replacement therapy or  ECMO  •To assess the effects of  IONIC Intervention vs.  Oseltamivir on the length  of hospital and intensive  care unit (ICU) stay  •To assess the effects of  IONIC Intervention vs.  Oseltamivir on the time  from treatment initiation to death. | 05/2021 | 07/2021 |
| NCT04973462 | Evaluation of The Efficacy of  Triazavirin Versus Oseltamivir in  Egyptian Patients Infected With  COVID-19 | Egypt | Recruiting | RCT | COVID-19 | 80 | 18-60 years | M/F | standard  treatment  COVID-19 (oseltamivir) Vs  Triazavirin | Interventional | 4 | •Physicians Global  Assessment to measure  the baseline COVID-19  signs and symptoms  •Time to recovery  •Incidence of re-detection of  viral RNA using PCR  •Incidence of Treatment-  Emergent Adverse Events  [Safety and Tolerability] | 08/01/2021 | 12/30/2021 |
| NCT04338698 | Hydroxychloroquine, Oseltamivir  and Azithromycin for the  Treatment of COVID-19  Infection: An RCT | Pakistan | Recruiting | RCT | COVID-19 | 500 | 18 years and older | M/F | HCQ  Vs  Oseltamivir  Vs  Azithromycin | Interventional | 3 | •Laboratory Result  •Clinical Outcome | 04/22/2020 | 11/30/2020 |
| NCT04255017 | A Prospective/  Retrospective,Randomized  Controlled Clinical Study of  Antiviral Therapy in the 2019-  nCoV Pneumonia | China | Recruiting | RCT | COVID-19 | 400 | 18 years and older | M/F | Abidol  hydrochloride  Vs  Oseltamivir  Vs  Lopinavir/ritonavir | Interventional | 4 | •Rate of disease remission  •Time for lung recovery  •Rate of no fever  •Rate of respiratory  symptom remission  •Rate of lung imaging  recovery  •Rate of  CRP,ES,Biochemical  Criterion (CK,ALT,Mb)  recovery  •Rate of undetectable viral RNA | 02/01/2020 | 07/01/2020 |
| NCT04261270 | A Randomized,Open,Controlled  Clinical Study to Evaluate  the Efficacy of ASC09F and  Ritonavir for 2019-nCoV  Pneumonia | China | Recruiting | RCT | COVID-19 | 60 | 18 -55 years | M/F | ASC09F  +Oseltamivir  Vs  Ritonavir  +Oseltamivir  Vs  Oseltamivir | Interventional | 3 | •Rate of comprehensive  adverse outcome  •Time of clinical remission  •Rate of no fever  •Rate of no cough  •Rate of no dyspnea  •Rate of no need for oxygen  inhalation  •Rate of undetectable viral  RNA  •Rate of mechanical  ventilation  •Rate of ICU admission  •Rate and time of  CRP,ES,Biochemical  criterion(CK,ALT,Mb)recovery | 02/01/2020 | 07/01/2020 |
| NCT04457609 | Administration of Allogenic UCMSCs  as Adjuvant Therapy for  Critically-Ill COVID-19 Patients | Indonesia | Recruiting | RCT | COVID-19 | 40 | 18 – 95 years | M/F | Oseltamivir  Vs  Azithromycin  Vs  Biological:  Umbilical Cord  Mesenchymal Stem  Cells | Interventional | 1 | •Clinical improvement:  Presence of dyspnea  •Clinical improvement:  presence of sputum  •Clinical improvement: fever  •Clinical improvement:  ventilation status  •Clinical improvement:  blood pressure  •Clinical improvement: heart  rate  •Clinical improvement:  respiratory rate  •Clinical improvement:  oxygen saturation  •General laboratory  outcome from leukocyte  level  •General laboratory  outcome from lymphocytes  level  •and 30 more | 07/2020 | 09/2020 |
| NCT04371601 | Safety and Effectiveness of  Mesenchymal Stem Cells in  the Treatment of Pneumonia of  Coronavirus Disease 2019 | China | Active, not  recruiting | RCT | COVID-19 | 60 | 18 – 70 years | M/F | Oseltamivir  Vs  hormones  Device: oxygen  therapy  Procedure:  mesenchymal stem  cells | Interventional | 1 | •Changes of oxygenation  index (PaO2/FiO2) ,blood  gas test  •Detection of TNF-# levels,  IL-10 levels  •Detection of immune cells  that secret cytokines,  including CXCR3+, CD4+,  CD8+, NK+ cells, and  regulatory T cells (CD4  + CD25 + FOXP3 + Treg  cells).  •Changes of c-reactive  protein and calcitonin | 03/01/2020 | 12/31/2022 |
| NCT02735707 | Randomized, Embedded,  Multifactorial Adaptive Platform  Trial for Community- Acquired  Pneumonia | USA, Australia | recruiting | RCT | Communityacquired  Pneumonia,  Influenza,COVID-19 | 7100 | 18 years and older | M/F | Fixedduration  Hydrocortisone  Shockdependent  hydrocortisone  Ceftriaxone  Moxifloxacin  or Levofloxacin  Piperacillintazobactam  Ceftaroline  Amoxicillinclavulanate  Macrolide  administered for  3-5 days  Macrolide  administered for up  to 14 days  Five-days  oseltamivir  and 20 more | Interventional | 4 | •All-cause mortality  •Days alive and not  receiving organ support in  ICU  •ICU Mortality  •ICU length of stay  •Hospital length of stay  •Ventilator free days  •Organ failure free days  •Health-related Quality of  life assessment  •Proportion of intubated  patients who receive a  tracheostomy  •Destination at time of  hospital discharge  •Readmission to the index  ICU during the index  hospitalization  •World Health Organisation  8-point ordinal scale  outcome | 04/11/2016 | 12/2023 |
